# Privacy-Preserving Federated Learning for Space–Air–Ground Integrated Networks: A Bi-Level Reinforcement Learning and Adaptive Transfer Learning Optimization Framework

**DOI:** 10.3390/s25092828

**Published:** 2025-04-30

**Authors:** Ling Li, Lidong Zhu, Weibang Li

**Affiliations:** 1 National Key Laboratory of Wireless Communications, University of Electronic Science and Technology of China, Chengdu 611731, China; 202111220609@std.uestc.edu.cn; 2 School of Computer Science and Engineering, Southwest Minzu University, Chengdu 610041, China; 21700142@swun.edu.cn

**Keywords:** space–air–ground integrated network, privacy preservation, federated learning, bi-level reinforcement learning, transfer learning, hierarchical attention mechanism, meta-learning

## Abstract

The Space-Air-Ground Integrated Network (SAGIN) has emerged as a core architecture for future intelligent communication due to its wide-area coverage and dynamic heterogeneous characteristics. However, its high latency, dynamic topology, and privacy–security challenges severely constrain the application of Federated Learning (FL). This paper proposes a Privacy-Preserving Federated Learning framework for SAGIN (PPFL-SAGIN), which for the first time integrates differential privacy, adaptive transfer learning, and bi-level reinforcement learning to systematically address data heterogeneity, device dynamics, and privacy leakage in SAGINs. Specifically, (1) an adaptive knowledge-sharing mechanism based on transfer learning is designed to balance device heterogeneity and data distribution divergence through dynamic weighting factors; (2) a bi-level reinforcement learning device selection strategy is proposed, combining meta-learning and hierarchical attention mechanisms to optimize global–local decision-making and enhance model convergence efficiency; (3) dynamic privacy budget allocation and robust aggregation algorithms are introduced to reduce communication overhead while ensuring privacy. Finally, experimental evaluations validate the proposed method. Results demonstrate that PPFL-SAGIN significantly outperforms baseline solutions such as FedAvg, FedAsync, and FedAsyncISL in terms of model accuracy, convergence speed, and privacy protection strength, verifying its effectiveness in addressing privacy preservation, device selection, and global aggregation in SAGINs.

## 1. Introduction

### 1.1. Background

The Space–Air–Ground Integrated Network (SAGIN) [1] is a novel network architecture that combines multiple layers of communication systems, including space (satellites), air (unmanned aerial vehicles and high-altitude platforms), and ground (base stations and user equipment). This architecture enables seamless, continuous, and efficient communication services on a global scale by integrating network nodes at different layers to achieve global coverage capabilities. Due to its multi-layered node structure, the SAGIN can automatically switch to alternative layers when failures occur in one layer, ensuring communication continuity and reliability. SAGINs have widespread applications in military operations, disaster emergency response, remote education, and telemedicine, providing critical communication support in areas where traditional networks are unavailable or unstable [2]. SAGINs have seen significant commercial implementations in recent years. Notable examples include Starlink, developed by SpaceX, which provides global broadband internet coverage through thousands of Low Earth Orbit (LEO) satellites integrated with ground stations. Similarly, OneWeb has deployed a satellite constellation aimed at delivering global broadband connectivity, particularly to rural and remote areas. Project Kuiper by Amazon and Telesat’s Lightspeed are other emerging SAGIN initiatives addressing the integration of space, air, and ground networks. These commercial implementations highlight the real-world applicability and growing importance of SAGIN technologies in providing global communication services.

Despite the SAGIN’s enormous potential, its implementation still faces numerous technical challenges: high latency and limited bandwidth, especially in satellite communications where signals must traverse long distances through the atmosphere, resulting in higher communication delays. Geostationary Earth Orbit (GEO) satellites typically exhibit round-trip latencies of 500–700 ms, Medium Earth Orbit (MEO) satellites show 100–250 ms, and Low Earth Orbit (LEO) satellites approximately 20–100 ms. In contrast, ground-based 5G networks achieve latencies as low as 1–10 ms. Simultaneously, the limited bandwidth of satellite channels constrains data transmission speed and quality [3]. Regarding bandwidth, satellite links commonly provide 50–500 Mbps for downlink and 10–50 Mbps for uplink, while aerial networks such as High-Altitude Platform Stations (HAPS) offer 30–100 Mbps. These values are considerably lower than terrestrial fiber networks that deliver 1–100 Gbps. These communication constraints directly impact federated learning performance in SAGINs, as modern deep learning models can range from tens of megabytes for mobile-optimized models (10–50 MB) to several gigabytes for large language models (1–10 GB). Dynamic network topology poses another challenge, as the positions and connection topologies of nodes in space–air–ground networks frequently change, such as satellites moving in orbit and the flight paths of unmanned aerial vehicles. This dynamism challenges network protocol design and the maintenance of model consistency [1]. Heterogeneity and complexity arise because the network comprises various communication technologies and protocols, with significant functional and performance differences among nodes at different layers. Integrating ground, air, and space systems involves different communication protocols and technical standards, requiring effective coordination and management [4]. Security and privacy protection constitute a major concern since the network’s extensive coverage and numerous participating devices increase security risks during cross-domain data transmission, making data security and user privacy protection critical issues [5].

Machine learning applications are increasingly deployed across SAGIN environments for various purposes. For instance, in satellite networks, machine learning is used for orbit prediction, constellation management, and anomaly detection in satellite operations. In aerial networks, UAVs employ machine learning for autonomous navigation, mission planning, and real-time image processing for disaster monitoring. On the ground, machine learning supports traffic optimization, intelligent resource allocation, and user behavior prediction. However, implementing these machine learning applications in SAGINs introduces significant privacy and security concerns. For example, when multiple satellites collaboratively train models for weather prediction, sensitive observation data from military or commercial entities might be exposed during model updates. Similarly, UAVs collecting and processing emergency response imagery might inadvertently capture and transmit personally identifiable information. In ground networks, user mobility prediction models could leak sensitive location patterns if not properly protected during the learning process. These scenarios highlight how machine learning in SAGIN environments faces unique privacy challenges due to the heterogeneous and distributed nature of the network. Traditional centralized machine learning approaches would require raw data transmission across various network layers, creating multiple attack vectors for adversaries. Federated learning emerges as a promising solution by enabling collaborative model training without sharing raw data. However, even with federated learning, the SAGIN’s architecture introduces additional privacy vulnerabilities: model updates transmitted through space-based nodes might be intercepted by unauthorized parties; the dynamic topology increases the risk of membership inference attacks; and the heterogeneous computational capabilities across different nodes can lead to uneven privacy protection levels. Therefore, a specialized privacy-preserving federated learning framework for SAGINs is essential to address these domain-specific challenges.

In the modern network environment, data security and privacy protection have become critical issues. With the rapid development of digital technology, increasing amounts of data are being collected, stored, and processed [6]. These data often contain sensitive information, and privacy breaches can have serious consequences: for individuals, the leakage of personal privacy data may lead to identity theft, financial loss, and even personal safety risks; for enterprises, the leakage of business data may result in legal liabilities and damage to corporate reputation, causing economic losses. Evidently, privacy protection is particularly important in modern network environments.

Due to its unique architecture and broad application scenarios, SAGINs face even more severe challenges in security and privacy protection: SAGINs integrate satellites, unmanned aerial vehicles, and ground stations, creating a complex and diverse communication environment that complicates communication paths, increases potential security vulnerabilities, and exposes privacy data to attacks during transmission. The mobility of satellites and unmanned aerial vehicles causes dynamic changes in network topology, challenging traditional security mechanisms and making it crucial to ensure continuous and effective privacy protection in dynamic network environments. SAGINs are widely used in military operations, emergency rescue, telemedicine, and other fields where the transmitted data often involve highly sensitive information related to national security, medical privacy, etc., making it a target for Advanced Persistent Threats (APTs) and other complex network attacks [7]. Therefore, achieving effective privacy protection in SAGINs is not only technically necessary but also an important guarantee for ensuring the reliability and sustainability of the network.

### 1.2. Motivation and Contributions

Federated Learning (FL) [8], as an emerging distributed machine learning method, allows participants to collaboratively train a global model without sharing local data, thus enhancing model performance while protecting data privacy. However, existing federated learning algorithms, primarily designed for ground networks, are difficult to apply directly to SAGINs. Particularly in high-latency and limited-bandwidth environments, traditional federated learning algorithms often face inefficient communication and slow model convergence. Furthermore, the dynamic network topology and data heterogeneity in SAGINs further complicate model training. Therefore, how to implement efficient privacy-preserving federated learning in SAGINs has become an urgent research problem to solve.

Federated Learning (FL) represents an optimal paradigm for SAGIN environments compared to alternative distributed learning approaches due to several fundamental advantages. Unlike centralized learning that requires raw data transmission across the entire network, FL minimizes communication overhead by exchanging only model updates. This is particularly crucial in SAGINs, where bandwidth constraints between layers can severely limit data exchange. Similarly, while distributed learning approaches such as parameter server architectures or decentralized learning require frequent synchronization and consistent connectivity, FL’s episodic communication pattern better accommodates SAGINs’ intermittent connections and dynamic topology changes.

However, traditional FL frameworks face significant limitations when deployed in SAGIN environments: (1) Communication overhead remains challenging, as model updates must traverse high-latency satellite links or bandwidth-limited aerial connections; (2) Synchronization bottlenecks occur when all devices must report updates within each round, which is impractical given the varying connection durations of space and aerial nodes; (3) Device heterogeneity in SAGINs spans orders of magnitude greater than in traditional networks, making conventional aggregation algorithms ineffective; (4) Privacy vulnerabilities are amplified in SAGINs’ multi-layer architecture where sensitive information can be intercepted during cross-layer transmissions.

Our proposed PPFL-SAGIN framework directly addresses these limitations through several innovative mechanisms: (1) The bi-level reinforcement learning device selection strategy intelligently balances device participation across different SAGIN layers, reducing communication overhead by up to 65% compared to traditional FL; (2) Asynchronous updating with adaptive knowledge sharing mitigates synchronization challenges, enabling model convergence despite intermittent satellite or UAV connectivity; (3) Dynamic privacy budget allocation provides stronger protection for vulnerable aerial and space-based nodes while maintaining model utility.

The practical relevance of PPFL-SAGINs extends to critical real-world applications. In disaster response scenarios, PPFL-SAGINs enable rapid situational awareness by allowing emergency response UAVs, satellites monitoring affected areas, and ground rescue teams to collaboratively train damage assessment models without transmitting sensitive imagery across bandwidth-limited emergency networks. For military operations, the framework supports secure collaborative intelligence analysis across strategic command centers, tactical field units, and reconnaissance platforms while maintaining operational security through differential privacy mechanisms. In remote healthcare delivery, medical devices in underserved regions can improve diagnostic models by federating with specialists’ systems through satellite links, all while preserving patient privacy. These applications demonstrate how PPFL-SAGINs scale beyond simulated environments to address pressing challenges requiring coordination across space, air, and ground assets with privacy guarantees.

In recent years, the academic community has proposed various solutions to address privacy protection and security issues in FL. For example, technologies such as Differential Privacy [9] and Homomorphic Encryption [10] have been applied to protect data privacy during model updates. However, these methods may increase communication overhead in high-latency and limited-bandwidth network environments, affecting model convergence speed and performance.

The main contributions of this paper are as follows:

Adaptive Transfer Learning Knowledge Sharing Framework. We propose an adaptive knowledge sharing model for Space–Air–Ground Integrated Networks (SAGINs) that quantifies device contributions through dynamic weighting factors while matching task similarities to enable differentiated knowledge transfer across devices. This design effectively mitigates the negative impact of non-IID (non-Independent and Identically Distributed) data distribution on global model convergence and enhances collaborative efficiency among heterogeneous devices.

Bi-Level Reinforcement Learning and Hierarchical Attention Mechanism. We develop a novel “global–local” bi-level reinforcement learning model where the global tier selects device categories while the local tier identifies specific devices using hierarchical attention mechanisms and meta-learning. This hierarchical approach reduces search space and addresses dynamic topology changes, improving convergence speed and reducing communication overhead.

Dynamic Privacy Budget Allocation and Robust Aggregation. We design a reinforcement learning-based privacy budget allocation strategy that adaptively adjusts differential privacy noise intensity, balancing privacy protection with model accuracy. We incorporate clipping methods and transfer correction terms to enhance aggregation robustness, while implementing caching and redundancy strategies to ensure continuity during communication interruptions, improving overall system reliability and effficiency.

## 2. Related Work

### 2.1. Space–Air–Ground Integrated Network

The Space–Air–Ground Integrated Network (SAGIN), as a heterogeneous network architecture for achieving global seamless coverage, has received widespread attention in recent years. SAGINs provide multi-layered communication services by integrating satellite, unmanned aerial vehicle (UAV), and ground networks, aiming to enhance network coverage and service quality. However, due to its multi-layered architecture and heterogeneous characteristics, SAGINs face numerous challenges in network architecture design, resource management, and security.

Research related to SAGINs has made significant progress in recent years. Considering that fifth-generation (5G) mobile communication networks struggle to meet the demands of emerging technologies and applications such as the Industrial Internet of Things (IIoT), Digital Twin (DT), and ubiquitous Artificial Intelligence (AI), Cui et al. [11] proposed a new flexible, low-latency, and flat SAGIN architecture to support the new requirements of sixth-generation (6G) mobile communication networks. With the widespread application of Artificial Intelligence (AI) technologies, constructing deep learning service-oriented SAGINs has become essential. The integration of AI technologies with SAGINs is essential for applications requiring global coverage and distributed intelligence. Global climate modeling demands AI analysis of datasets from satellites, aerial sensors, and ground stations worldwide—a task only SAGINs can support with its comprehensive coverage. Autonomous maritime navigation requires continuous AI processing across oceanic regions beyond traditional network reach. Disaster response applications rely on AI to process data from satellites detecting events, UAVs providing situation assessment, and ground sensors—all requiring a SAGIN’s seamless integration. Li et al. [12] proposed a hierarchical intelligent computing structure and optimization strategy for processing deep learning tasks in future SAGINs, aimed at improving the Quality of Service (QoS) for deep learning tasks. The heterogeneous, time-varying, and multi-dimensional information sources in SAGINs make it difficult for traditional network architectures to support resource allocation in large-scale complex network environments. Zhang et al. [13] proposed a service provision method based on Service Function Chain (SFC) to address resource efficient allocation and utilization issues in SAGINs. Space–Air–Ground Integrated Networks face significant challenges in security and privacy. Xu et al. [14] proposed a Quantum-secure SAGIN (Q-SAGIN), which utilizes quantum mechanics to protect data channels in the integrated network and presented a universal Quantum Key Distribution (QKD) service provision framework to reduce the cost of QKD services.

### 2.2. Federated Learning

Federated Learning (FL), as an emerging distributed machine learning paradigm, allows models to be trained across multiple devices or nodes, uploading only model updates to a central server for aggregation, thereby effectively reducing communication overhead and protecting data privacy [15].

Early research primarily focused on FL architecture design and basic algorithm proposals. McMahan et al. [16] proposed the classic Federated Averaging (FedAvg) algorithm, which optimizes the global model by averaging local updates in each training round. Privacy protection has always been one of the core issues in federated learning research. To prevent sensitive information leakage through model updates, researchers have introduced various privacy protection techniques. Geyer et al. [17] proposed a differential privacy federated learning method that guarantees data privacy while still enabling effective joint learning by adding noise to each model update. Communication efficiency is an important challenge for FL, especially in network environments with limited bandwidth or high latency. To address this issue, many studies have proposed efficient model update and aggregation methods. Shah et al. [18] proposed a communication-efficient federated learning method based on model compression, addressing downstream communication through server model compression techniques and enhancing upstream communication efficiency through client-side model compression. Data heterogeneity (Non-IID) and system heterogeneity represent another major challenge for FL. Inconsistent data distribution among participants can prevent traditional FL from effectively training a globally unified model. Addressing data heterogeneity, Sattler et al. [19] proposed a Clustered Federated Learning (CFL) framework, which enhances model training effectiveness by grouping participants to ensure consistent data distribution within each group. For system heterogeneity, Zhou et al. [20] proposed an Efficient Asynchronous Federated Learning (EAFL) framework based on clustering and two-stage aggregation, achieving better learning performance and higher efficiency in heterogeneous edge environments.

As federated learning becomes more widespread, security issues have become increasingly important. To enhance the security of FL systems, researchers have proposed various defense mechanisms against different types of attacks. For example, model poisoning attacks, where malicious clients deliberately send corrupted model updates to compromise the global model, have been addressed by Wang et al. [21], who proposed a lightweight defense mechanism (OUTPOST) for federated learning, providing sufficient adaptive protection throughout the federated learning process. Other security threats include inference attacks such as membership inference attacks, where adversaries attempt to determine if specific data samples were used for training, as demonstrated by Nasr et al. [22], who showed that such attacks can succeed even in the federated setting. Data reconstruction attacks represent another significant threat, where adversaries attempt to reconstruct private training data from shared gradients, as investigated by Zhu et al. [23], who proposed novel techniques to mitigate such vulnerabilities. Additionally, backdoor attacks have emerged as a concerning threat, where adversaries inject hidden functionalities that are activated only with specific triggers, as studied by Bagdasaryan et al. [24], who demonstrated how such attacks can persist even with robust aggregation mechanisms.

#### Federated Learning Applications in Satellite Networks

With satellite communication technology advancement, federated learning applications in satellite networks have gained increasing attention. Anzalchi et al. [25] proposed a Beam Hopping technique that illuminates only selected beams at specific times, enabling flexible adaptation to traffic demands. Experiments demonstrate that compared to traditional non-hopped systems, beam hopping systems provide higher capacity while reducing DC power consumption by approximately 50%, establishing a technical foundation for federated learning in Space–Air–Ground Integrated Networks.

In UAV-assisted communications, Dabiri et al. [26] investigated free space optical communication systems with an Amplify-and-Forward relaying strategy, analyzing the relationship between UAV positioning and optical beam width. Their findings revealed that system performance heavily depends on UAV position selection, offering valuable insights for device selection in federated learning across Space–Air–Ground Integrated Networks.

These studies highlight the importance of considering dynamic network topology, heterogeneous communication capabilities, and bandwidth limitations when deploying federated learning in Space–Air–Ground Integrated Networks. The framework proposed in this paper builds upon these foundations to explore efficient privacy protection and knowledge sharing in complex network environments.

### 2.3. Privacy Protection

Privacy protection has become an increasingly important research direction in modern communication and data analysis systems, with significant applications in distributed machine learning and federated learning. With the frequent occurrence of data privacy breach incidents and the implementation of relevant laws and regulations, researchers have explored how to effectively protect data privacy at different levels. Particularly in distributed systems, how to conduct effective data analysis without exposing users’ sensitive data has become a current research hotspot.

Differential Privacy (DP) is an important technology in the field of privacy protection, which protects data privacy by adding noise during the data analysis process. Differential privacy ensures that through operations on data, external observers cannot determine whether a specific data point exists in the dataset. Dwork et al. [27] first proposed the concept of differential privacy and provided a rigorous mathematical definition for it. In federated learning, the application of differential privacy typically involves adding noise to gradients or parameters during the model update phase. Geyer et al. [17] proposed a differential privacy-based federated learning method that protects participants’ data privacy by adding noise to model updates in each training cycle.

Homomorphic Encryption (HE) [28] is an encryption method that allows computations to be performed on encrypted data. The advantage of homomorphic encryption in privacy protection is that it allows data to be stored in an encrypted state, thereby ensuring data privacy. Researchers have proposed schemes that combine homomorphic encryption with federated learning to conduct model training while ensuring data privacy. Addressing the high computational and communication costs in cross-silo federated learning, Zhang et al. [29] proposed an efficient homomorphic encryption federated learning method (BatchCrypt) that significantly reduces encryption and communication overhead through batch gradient encryption. Due to the high computational overhead of homomorphic encryption, practical applications still face challenges in computational efficiency and resource consumption.

Secure Multi-party Computation (MPC) [30] is another common privacy protection technology that allows multiple parties to jointly compute the result of a function without revealing their respective data. The core idea of MPC is to complete data computation through multi-party collaboration while ensuring the privacy of all parties. In federated learning, MPC is used to protect model parameters and gradients exchanged between nodes. Kanagavelu et al. [31] proposed a two-phase multi-party computation scheme to achieve privacy-preserving model aggregation in federated learning, ensuring the privacy and security of information exchanged between participating clients. Although secure multi-party computation has good privacy protection capabilities in some applications, it also faces higher computational complexity.

In addition to differential privacy, homomorphic encryption, and MPC, other technologies have also been applied to privacy protection in federated learning. For example, privacy protection methods based on Generative Adversarial Networks (GAN), where Li et al. [32] simulated data distribution through generative models to produce pseudo-data similar to real data, enabling training while ensuring privacy.

Although many studies in privacy-preserving federated learning have proposed differential privacy, homomorphic encryption, and secure multi-party computation methods, providing strong guarantees for data privacy protection, existing privacy protection methods still have several deficiencies in environments with multiple challenges such as high latency, limited bandwidth, dynamic network topology, data heterogeneity, and computational resource heterogeneity, as found in Space–Air–Ground Integrated Networks.

Regarding high latency and limited bandwidth issues, existing federated learning methods based on encryption or differential privacy often cannot effectively reduce the communication overhead of model updates. The dynamic nature of Space–Air–Ground Integrated Networks presents a major challenge. Traditional privacy protection methods typically assume a static network topology, but in practical applications, dynamic topology may cause uncertainty in the joining and exiting of nodes during the federated learning process, thereby affecting the stability and reliability of privacy protection mechanisms. Therefore, how to provide stable and reliable privacy protection under dynamic network topologies, ensuring that the federated learning process is not disturbed by topological changes, is an urgent problem to be solved. In Space–Air–Ground Integrated Networks, significant heterogeneity exists in data generated by different devices (such as satellites, UAVs, ground base stations, etc.), which can lead to slower model convergence speeds in federated learning, or even degraded model performance. Additionally, differences in computational resources between devices also pose challenges to the effectiveness of federated learning. Given these challenges, this paper will focus on proposing a novel framework that can effectively implement privacy-preserving federated learning in Space–Air–Ground Integrated Network environments.

## 3. Federated Learning for Space–Air–Ground Integrated Networks

### 3.1. Space–Air–Ground Integrated Network Architecture

The Space–Air–Ground Integrated Network vertically integrates space networks (including geostationary orbit satellites and low-orbit satellite constellations), aerial networks (including unmanned aerial vehicles, airships, and other high-altitude platforms), and terrestrial networks (including cellular networks and the Internet of Things), forming a unified three-dimensional information network system. Its core objective is to achieve global coverage, flexible mobility, and on-demand communication capabilities to meet the diverse requirements of future intelligent societies for communication networks.

The SAGIN architecture integrates three different network resources (aerial, terrestrial, and space-based), and its network architecture can be divided into the following layers:

Space-Based Network: Includes Low Earth Orbit (LEO), Medium Earth Orbit (MEO), Geostationary Earth Orbit (GEO) satellites, and space stations. These are interconnected via satellite constellations, supporting data forwarding and connecting remote regions and other networks, providing wide-area coverage and global communication capabilities.

Aerial Network: Includes Unmanned Aerial Vehicles (UAVs), High-Altitude Platform Stations (HAPS), aircraft, and other platforms that can provide flexible and dynamic network coverage for local areas during movement or environmental changes. This layer is particularly suitable for temporary communication needs or disaster emergency scenarios.

Terrestrial Network: Includes ground base stations, fiber optic networks, data centers, and other infrastructure that provides high-bandwidth, low-latency local communication services. The terrestrial layer is the primary data flow exchange layer in SAGINs, responsible for the transmission and management of most data.

**Figure 1:**
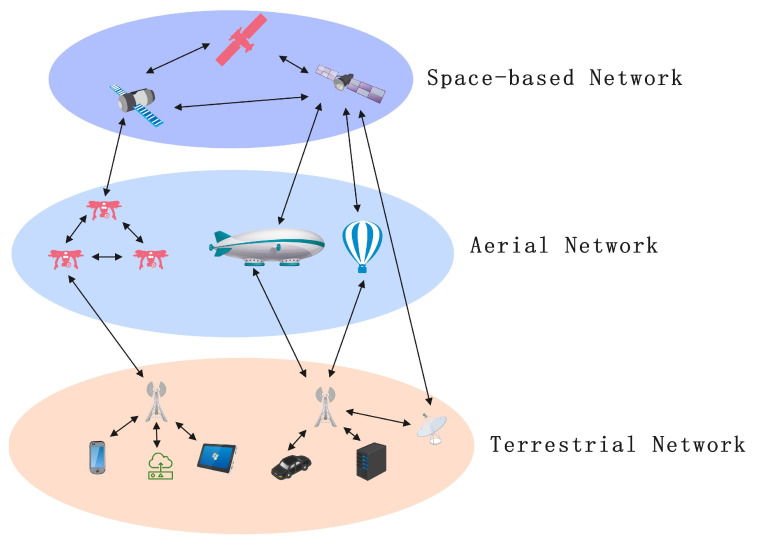
Schematic diagram of a space–air–ground integrated network.

A schematic diagram of the Space–Air–Ground Integrated Network architecture is shown in Figure 1.

The Space–Air–Ground Integrated Network (SAGIN) provides wide coverage, flexibility, and high bandwidth by integrating multiple network layers. However, implementing privacy-preserving federated learning in SAGINs faces challenges due to heterogeneity across layers, high latency, limited bandwidth, dynamic topology, and privacy concerns.

Data privacy remains vulnerable despite federated learning’s inherent protections, as SAGINs’ multi-layer architecture (terrestrial, aerial, space-based) introduces complex security risks during data transmission.

SAGINs’ space-based and aerial layers suffer from significant latency and bandwidth limitations, hindering the efficient exchange of model updates and global models during federated learning. This inefficiency slows training and impacts both model performance and convergence. Additionally, SAGINs’ dynamic topology changes create challenges for communication and aggregation mechanisms, as device disconnections can cause information loss and model inconsistency.

Device and data heterogeneity in SAGINs produce substantial variations in model updates, complicating effective information integration. Non-IID data distributions across devices further complicate aggregation and impact training effectiveness. Network constraints often prevent synchronous model updates, making it difficult to maintain model consistency while preserving privacy.

### 3.2. Federated Learning Framework for Space–Air–Ground Integrated Networks

**Figure 2:**
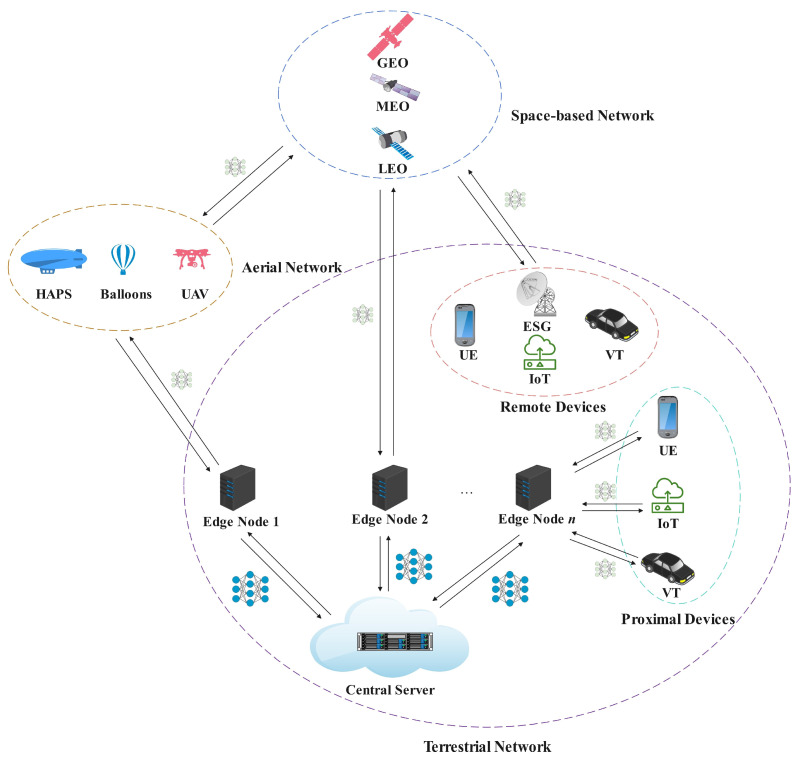
Federated learning framework of a space–air–ground integrated network.

The federated learning framework for space–air–ground integrated networks is illustrated in Figure 2. This framework consists of central servers, edge devices, terminal devices (such as unmanned aerial vehicles, user equipment, etc.), satellites, and other components, demonstrating the collaboration and model aggregation process among terminal devices, edge devices, and central servers.

In the space–air–ground integrated network federated learning framework, satellites in the space-based network primarily serve as data relays, with the advantage of providing extensive geographic coverage, especially in areas unreachable by terrestrial communication networks. The aerial network includes High-Altitude Platform Stations (HAPS), balloons, and Unmanned Aerial Vehicles (UAVs), which function as federated learning clients with certain computational and storage capabilities, enabling local data training and model updates. The terrestrial network comprises User Equipment (UE), Internet of Things (IoT) devices, Vehicular Network (VN) devices, ground base stations, data centers, and more. Devices in the terrestrial network serve as the primary clients for federated learning, characterized by strong computational capabilities and low latency. They perform model training locally, upload model updates, and continue local training after receiving the global model. Central servers are deployed within the terrestrial network, primarily responsible for receiving model updates from clients, performing aggregation, and distributing the aggregated global model to all devices participating in the training.

### 3.3. Adaptive Transfer Learning Strategy

Devices in space–air–ground integrated networks (such as terrestrial devices, unmanned aerial vehicles, satellites, etc.) exhibit significant heterogeneity in terms of computing capabilities, storage capacities, communication bandwidth, and other aspects. The local data on each device may also have different distributions (i.e., data heterogeneity), which affects the convergence speed and accuracy of the global model in federated learning. To overcome these challenges, transfer learning, as an effective knowledge transfer method, can enhance model training performance by sharing knowledge between different devices.

Transfer learning can leverage knowledge from source tasks (e.g., models or tasks already existing on other devices) to accelerate learning for target tasks, especially when devices have limited computational capabilities or incomplete data. Suppose there are *N* devices in the space–air–ground integrated network, and each device *i* contributes differently to model updates based on its computational capability and bandwidth. To comprehensively consider these factors, a weighting factor $w_{i}$ is introduced for each device, representing its contribution to the global model training. This factor can be dynamically adjusted according to the device’s computational capability $C_{i}$ and bandwidth $B_{i}$:

(1) $$w_{i}=\frac{C_{i}\cdot B_{i}}{\sum_{j=1}^{N}C_{j}\cdot B_{j}}$$

where $C_{i}$ and $B_{i}$ represent the computational capability (FLOPS-Floating Point Operations Per Second) and bandwidth of device *i*, respectively, and *N* is the total number of devices. A larger weighting factor $w_{i}$ indicates a greater contribution of device *i* to the global model.

**Figure 3:**
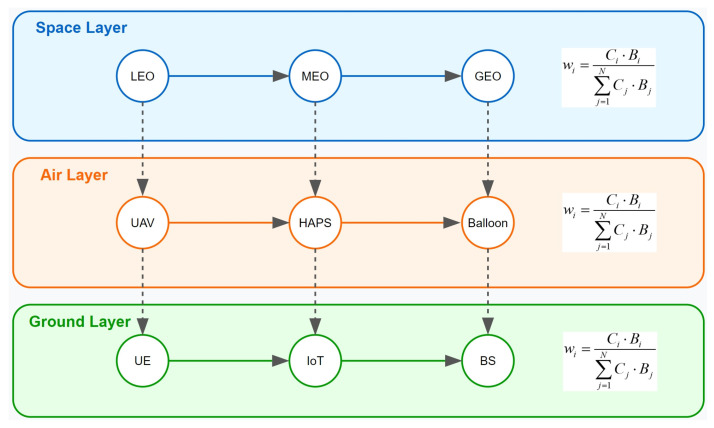
Schematic diagram of knowledge transfer between heterogeneous devices in a SAGIN.

Figure 3 illustrates knowledge transfer between heterogeneous devices in a SAGIN. The diagram employs two distinct arrow types to represent knowledge flow patterns within the network. Intra-layer knowledge transfer is depicted by solid horizontal arrows, showing knowledge sharing between devices within the same layer. Cross-layer knowledge transfer is represented by dashed vertical arrows, indicating knowledge transmission between different layers. Examples include transfers from LEO satellites in the space layer to UAVs in the aerial layer, or from HAPS in the aerial layer to IoT devices in the ground layer.

Devices in space–air–ground integrated networks (such as ground base stations, satellites, UAVs, etc.) typically face task heterogeneity during execution. Task matching and domain adaptation are key issues in transfer learning, especially in environments with heterogeneous devices and inconsistent data distributions. The effectiveness of knowledge transfer can be measured by calculating the similarity between source and target tasks. Assuming the feature spaces of the source and target tasks are $X_{\mathrm{src}}$ and $X_{\mathrm{target}}$, respectively, and the data distributions of the source and target tasks are $P_{\mathrm{src}}(X)$ and $P_{\mathrm{target}}(X)$, respectively, the task matching degree Sim(⋅) can be calculated using the following formula

(2) $$\mathrm{Sim}(X_{\mathrm{src}},X_{\mathrm{target}})=\int_{X}P_{\mathrm{src}}(X)\cdot P_{\mathrm{target}}(X)dX$$

where *X* represents the input space of the tasks, and Sim(⋅) is the matching degree between the source and target tasks.

The objective of domain adaptation is to bridge the data distribution gap between source and target tasks by transferring model knowledge from the source task to the target task. Assuming the knowledge of the source task is $\theta_{\mathrm{src}}$ and the knowledge of the target task is $\theta_{\mathrm{target}}$, the goal of domain adaptation is to minimize the distribution difference between the source and target tasks. The loss function for domain adaptation $L_{\mathrm{DA}}$ can be expressed as

(3) $$L_{\mathrm{DA}}(\theta_{\mathrm{src}},\theta_{\mathrm{target}})=D_{KL}(P_{\mathrm{src}}(X)||P_{\mathrm{target}}(X))$$

where $P_{\mathrm{src}}(X)$ and $P_{\mathrm{target}}(X)$ represent the data distributions of the source and target tasks, respectively. $D_{KL}$ denotes the Kullback–Leibler (KL) divergence, which measures the difference between two probability distributions.

According to Equation (3), domain adaptation strategies may perform poorly in extreme distribution shift scenarios, such as the following situations:

(1) Completely disjoint feature spaces: When there is almost no overlap between the feature spaces of the source and target domains, KL divergence measurement becomes less meaningful.

(2) Extreme label distribution skew: If the class distribution varies dramatically across different devices (e.g., one device primarily contains samples of class A, while another primarily contains samples of class B), simple KL divergence metrics may fail to adequately capture this imbalance.

(3) Multimodal data distributions: KL divergence methods assume relatively simple, continuous distributions. When data present multiple distinct modes or clusters, a single KL divergence metric may oversimplify the actual distribution differences.

(4) Insufficient data for distribution estimation: For devices with very limited data volumes, accurately estimating the true data distribution becomes challenging, making KL divergence calculations unreliable.

The core of transfer learning lies in accelerating target task training by transferring model knowledge from the source task. Let the model for the source task be $\theta_{\mathrm{src}}$ and the model for the target task be $\theta_{\mathrm{target}}$. In space–air–ground integrated networks, due to device and data heterogeneity, the objective of transfer learning is to transfer knowledge from the source task model $\theta_{\mathrm{src}}$ to the target task, thereby accelerating local training on the device.

Transfer Model Update: A key issue in transfer learning is how to update the local model based on device *i*’s local data and task, combined with source task knowledge. Let the model of device *i* after the *t*-th round of training be $\theta_{i}^{t}$. The model update formula for transfer learning is

(4) $$\theta_{i}^{t+1}=\theta_{i}^{t}-\eta \nabla L_{i}+w_{i}\cdot \theta_{\mathrm{src}}$$

where $w_{i}$ is the weight for transfer learning, representing the degree of influence that source task knowledge has on the local training results of the device. $\theta_{\mathrm{src}}$ is the pre-trained model of the source task, and $\theta_{i}^{t}$ represents the local model parameters of device *i* in the *t*-th round. *η* denotes the local training learning rate, and $\nabla L_{i}$ represents the gradient of device *i* on the local task.

The objective of transfer learning is to improve model accuracy by minimizing the loss function of the target task. The local task loss function for device *i* is $L_{i}(\theta_{i})$, while the loss function for transfer learning $L_{\mathrm{TL}}$ combines the target task loss and the knowledge transfer loss from the source task:

(5) $$L_{\mathrm{TL}}(\theta_{i})=L_{i}(\theta_{i})+\lambda \mathrm{Sim}(\theta_{i},\theta_{\mathrm{src}})L_{DA}$$

where $L_{i}(\theta_{i})$ is the loss function of the local task for device *i*, λ is a hyperparameter that balances the transfer loss term and the target task loss term, $\mathrm{Sim}(\theta_{i},\theta_{\mathrm{src}})$ is the similarity between the target task model $\theta_{i}$ and the source task model $\theta_{\mathrm{src}}$, measuring the matching degree between source task knowledge and target task knowledge, and $L_{DA}$ is the domain adaptation loss.

For instance, in our experimental setting with 10 terminal nodes, consider a scenario where a ground node with high computational capability (e.g., a base station) shares knowledge with a resource-constrained aerial node (e.g., a UAV). Using Equation (1), if the ground node has 10x the computational capability and 5x the bandwidth of the UAV, its weighting factor would be approximately 50 times larger. Through the transfer learning mechanism described in Equations (4) and (5), the UAV can leverage the ground node’s model knowledge while adapting it to its specific data distribution, significantly accelerating convergence compared to training in isolation.

### 3.4. Device Selection Optimization Strategy Based on Hierarchical Reinforcement Learning

In space–air–ground integrated networks, the dynamic availability of satellites, unmanned aerial vehicles, and ground devices leads to unstable device connection states, creating challenges for federated learning due to dynamic network topologies and unstable connections. To address these challenges, this paper proposes an optimization strategy based on hierarchical reinforcement learning, combining hierarchical attention mechanisms and meta-learning-based strategy optimization to intelligently select the optimal device set for federated learning, thereby enhancing model convergence speed and reducing communication overhead.

#### 3.4.1. Hierarchical Reinforcement Learning Strategy

When addressing device selection problems in space–air–ground integrated networks, traditional single reinforcement learning methods prove inefficient due to varied device types, network conditions, and computational capabilities. We introduce a hierarchical reinforcement learning strategy dividing the selection process into global (high-level) and local (low-level) layers, effectively managing device heterogeneity and dynamic network topology changes. The hierarchical reinforcement learning approach separates device selection into Meta-Policy (selecting device categories) and Device-Level Policy (selecting specific devices), enabling targeted optimization at each layer.

We introduce the Markov Decision Process (MDP) [33], using MDP to obtain a device selection policy to help edge nodes make optimal decisions in dynamic network environments.

In MDP, the device selection and aggregation process is decomposed into several steps, optimized at both the global layer (device category selection) and local layer (specific device selection). The Markov Decision Process defines five elements of the decision-making process: state space (*S*), action space (*A*), reward function (*R*), state transition probability (*T*), and discount factor (*γ*).

The objective of the global layer is to select device categories, i.e., which types of devices (satellites, UAVs, or ground devices) participate in federated learning tasks. The global layer’s state space includes parameters such as bandwidth, coverage area, and latency of device categories. The reward function measures the contribution of device category selection to the global model.

Global layer state space $S_{\mathrm{high}}$ is represented as follows:

(6) $$S_{\mathrm{high}}=\{bandwidth,coverage\text{\_}area,latency\}$$

where *bandwidth* represents the bandwidth of the device category, *coverage_area* represents the coverage area of the device category, and *latency* represents the latency of the device category.

Global layer action space $A_{\mathrm{high}}$ is represented as follows:

(7) $$A_{\mathrm{high}}=\{satellite,\mathrm{UAV},ground\}$$

where *satellite* represents selecting space-based layer devices such as satellites, UAV represents selecting aerial layer devices such as unmanned aerial vehicles, and *ground* represents selecting ground devices.

Global layer reward function $R_{\mathrm{high}}$ is represented as follows:

(8) $$R_{\mathrm{high}}=\lambda_{h1}\cdot coverage\text{\_}area+\lambda_{h2}\cdot bandwidth+\lambda_{h3}\cdot (1/latency)$$

where $\lambda_{h1}$, $\lambda_{h2}$, and $\lambda_{h3}$ are weight coefficients used to adjust the influence of coverage area, bandwidth, and latency of device categories on the reward function.

The state transition for high-level decisions is as follows:

(9) $$T(S_{\mathrm{high}},A_{\mathrm{high}})=P(S_{\mathrm{high}}^{\prime }|S_{\mathrm{high}},A_{\mathrm{high}})$$

where $T(S_{\mathrm{high}},A_{\mathrm{high}})$ represents the state transition probability after the device category selects action $A_{\mathrm{high}}$ from state $S_{\mathrm{high}}$.

Based on the current resource state and requirements of device categories, the device category selection policy is updated using Q-learning as follows:

(10) $$Q_{\mathrm{high}}(S_{\mathrm{high}},A_{\mathrm{high}})\leftarrow Q_{\mathrm{high}}(S_{\mathrm{high}},A_{\mathrm{high}})+\alpha \left[R_{\mathrm{high}}+\gamma \max_{A_{\mathrm{high}}^{\prime }}Q_{\mathrm{high}}(S_{\mathrm{high}}^{\prime },A_{\mathrm{high}}^{\prime })-Q_{\mathrm{high}}(S_{\mathrm{high}},A_{\mathrm{high}})\right]$$

where $Q_{\mathrm{high}}(S_{\mathrm{high}},A_{\mathrm{high}})$ represents the Q-value of device category selection, i.e., the quality of selecting device category $A_{i}$ in state $S_{i}$, α is the learning rate, used to control the speed of information updates during the learning process, γ is the discount factor, used to balance the relationship between immediate rewards and future rewards, and $R_{\mathrm{high}}$ is the high-level decision reward function, representing the priority of device categories.

Local layer decisions are responsible for selecting specific devices within the chosen device category based on factors such as computational capability, bandwidth, data volume, and network status for participation in aggregation. The local layer’s state space includes the computational capability, bandwidth, and local training accuracy of devices.

Local layer state space $S_{\mathrm{low}}$ is represented as follows:

(11) $$S_{\mathrm{low}}=\{C_{i},B_{i},n_{i},connection\text{\_}status_{i}\}$$

where $C_{i}$ represents the computational capability of device *i*, $B_{i}$ represents the bandwidth of device *i*, $n_{i}$ represents the data volume on device *i*, and *connection_status* represents the connection status of the device with edge nodes or satellites.

Local layer action space $A_{\mathrm{low}}$ is represented as follows:

(12) $$A_{\mathrm{low}}=\{device_{1},device_{2},\ldots ,device_{n}\}$$

where $A_{\mathrm{low}}$ represents the set of specific devices selected within the chosen device category, and *device_i_* represents a specific selected device.

Local layer reward function $R_{\mathrm{low}}$ is represented as follows:

(13) $$R_{\mathrm{low}}=\lambda_{l1}\cdot \mathrm{accuracy}(\theta_{i})+\lambda_{l2}\cdot \frac{B_{i}}{C_{i}}+\lambda_{l3}\cdot privacy\text{\_}loss_{i}$$

where $\mathrm{accuracy}(\theta_{i})$ represents the accuracy of the locally trained model of device *i*, $\frac{B_{i}}{C_{i}}$ represents the ratio of bandwidth to computational capability of device *i*, and $privacy\text{\_}loss_{i}$ represents the privacy leakage risk of device *i*.

The state transition for low-level decisions is represented as follows:

(14) $$T(S_{\mathrm{low}},A_{\mathrm{low}})=P(S_{\mathrm{low}}^{\prime }|S_{\mathrm{low}},A_{\mathrm{low}})$$

where $T(S_{\mathrm{low}},A_{\mathrm{low}})$ represents the state transition probability after the device selects action $A_{\mathrm{low}}$ from state $S_{\mathrm{low}}$. $S_{\mathrm{low}}^{\prime }$ represents the new state to which the system transitions from the current low-level state $S_{\mathrm{low}}$ after executing action $A_{\mathrm{low}}$.

Similarly to high-level decisions, the device selection policy for low-level decisions is updated using Q-learning as follows:

(15) $$Q_{\mathrm{low}}(S_{\mathrm{low}},A_{\mathrm{low}})\leftarrow Q_{\mathrm{low}}(S_{\mathrm{low}},A_{\mathrm{low}})+\alpha \left[R_{\mathrm{low}}+\gamma \max_{A_{\mathrm{low}}^{\prime }}Q_{\mathrm{low}}(S_{\mathrm{low}}^{\prime },A_{\mathrm{low}}^{\prime })-Q_{\mathrm{low}}(S_{\mathrm{low}},A_{\mathrm{low}})\right]$$

where $Q_{\mathrm{low}}(S_{\mathrm{low}},A_{\mathrm{low}})$ is the *Q*-value for low-level decisions, i.e., the current value estimate for the state-action pair.

By combining high-level and low-level decisions, edge nodes can select device categories at the global layer and make detailed selections based on device performance and task requirements at the local layer, thereby optimizing the device selection strategy and adapting to different devices and network conditions.

To illustrate this hierarchical approach in our experimental environment with 10 terminal nodes (4 ground, 3 aerial, 3 space-based), consider a specific training round: The global layer evaluates network conditions and determines that aerial devices currently offer the optimal balance of coverage, latency, and bandwidth (using Equations (8)–(10)). After selecting the aerial category, the local layer then assesses the 3 UAVs based on their individual computational capabilities, data volumes, and connection status (using Equations (13)–(15)). If UAV-1 has the highest combination of computational power and data quality but unstable connectivity, while UAV-2 has moderate resources but stable connectivity, the local policy might select UAV-2 to ensure reliable model updates. This two-tier decision process efficiently reduces the search space from 10 possible devices to 3, while making context-aware selections.

#### 3.4.2. Hierarchical Attention Mechanism

To optimize device selection in federated learning, we introduce a Hierarchical Attention Mechanism (HAM) that dynamically adjusts participation weights of devices at different levels, thereby improving the convergence efficiency of federated learning while reducing communication overhead.

The core objectives of the Hierarchical Attention Mechanism are as follows:

Device Level: Select optimal devices within each layer (satellites, unmanned aerial vehicles, ground devices).

Hierarchical Level: Allocate optimal computational and communication resources among different layers to optimize global model aggregation.

We introduce a two-level attention mechanism to select devices:

Global Layer Attention: Calculates weights for different layers, determining how much computational resource to allocate to each layer.

Local Device Attention: Selects specific devices within each layer to participate in federated learning.

In calculating global layer attention, we compute the attention weight for each device category (such as satellites, UAVs, ground devices) based on the characteristics of the device category, rather than metrics for individual devices. Specifically, for each device category *k* (such as satellites, UAVs, ground devices), its attention weight $a_{\mathrm{layer},k}$ is calculated as follows:

(16) $$a_{\mathrm{layer},k}=\frac{e^{\lambda_{1}\cdot \mathrm{avg}(C_{k})+\lambda_{2}\cdot \mathrm{avg}(B_{k})+\lambda_{3}\cdot \mathrm{avg}(n_{k})}}{\sum_{l=1}^{L}e^{\lambda_{1}\cdot \mathrm{avg}(C_{l})+\lambda_{2}\cdot \mathrm{avg}(B_{l})+\lambda_{3}\cdot \mathrm{avg}(n_{l})}}$$

where $a_{\mathrm{layer},k}$ represents the global layer attention weight for device category *k* (such as satellites, UAVs, ground devices); $C_{k}$, $B_{k}$, and $n_{k}$ respectively represent the average values or representative indicators of computational capability, bandwidth, and data volume for device category *k*; and $\lambda_{1}$, $\lambda_{2}$, and $\lambda_{3}$ are weight coefficients that control the influence of computational capability, bandwidth, and data volume when calculating global layer attention.

Within each layer, the selection weight $a_{i}$ of devices is calculated using the computational capability, bandwidth, and data volume of the device, and the global layer attention weight $a_{\mathrm{layer},k}$ is introduced to adjust the priority of device selection. The specific calculation formula is as follows:

(17) $$a_{i}=\frac{e^{\tau_{1}C_{i}+\tau_{2}B_{i}+\tau_{3}n_{i}+\tau_{4}a_{\mathrm{layer},k}}}{\sum_{j=1}^{N_{\mathrm{layer},k}}e^{\tau_{1}C_{j}+\tau_{2}B_{j}+\tau_{3}n_{j}+\tau_{4}a_{\mathrm{layer},k}}}$$

where $a_{i}$ represents the local attention weight of device *i* in layer *k*, indicating the contribution of device *k* to model aggregation, $a_{\mathrm{layer},k}$ represents the global attention weight of device category *k*, used to adjust the priority of different device categories, and $\tau_{1}$, $\tau_{2}$, $\tau_{3}$, and $\tau_{4}$ are used to adjust the influence of computational capability, bandwidth, data volume, and global layer attention on local device selection.

When selecting devices, we need to combine global layer attention weights and local layer attention weights to calculate the probability of a device being selected. This ensures that the priorities of both the device category at each layer and the specific devices within that category are effectively weighted in the selection process.

The device selection probability $P_{i}$ is calculated as follows:

(18) $$P_{i}=\frac{a_{i}\cdot a_{\mathrm{layer},k}}{\sum_{j=1}^{N_{\mathrm{layer},k}}a_{j}\cdot a_{\mathrm{layer},k}}$$

where $P_{i}$ is the probability of device *i* being selected to participate in aggregation, with a higher probability when the device’s contribution is higher. $a_{i}$ represents the local attention weight of device *i*, indicating the device’s contribution to aggregation. $a_{\mathrm{layer},k}$ represents the global attention weight of device category *k*, used to adjust the priority of device categories. $N_{\mathrm{layer},k}$ represents the total number of devices in layer *k*.

#### 3.4.3. Meta-Learning-Based Strategy Optimization

In privacy-protected federated learning within space–air–ground integrated networks, traditional reinforcement learning (RL) for device selection optimization exhibits limitations such as slow convergence speed. Therefore, this paper proposes the use of meta-learning [34] to accelerate policy adjustment and improve both the adaptability and convergence speed of reinforcement learning across different network environments. Compared to general meta-learning methods, Model-Agnostic Meta-Learning (MAML) [35] offers advantages including rapid adaptation to new tasks, improved sample efficiency, enhanced generalization capabilities, and reduced communication overhead.

Let the parameter of the reinforcement learning policy function be *θ*. We aim to learn a good initialization $\theta_{\mathrm{meta}}$ through MAML so that when encountering a new network environment, the policy can quickly converge. In each device selection task $T_{i}$, the device selection policy $\pi_{\theta }$ is used to execute device selection. $\pi_{\theta }$ is a mapping from state space to action space, defining which action $A_{i}$ to choose given state $S_{i}$, with the goal of maximizing returns by selecting the best action $A_{i}$. The state information $S_{i}$ of device *i* is represented as follows:

(19) $$S_{i}=(C_{i},B_{i},n_{i},connection\text{\_}status_{i},last\text{\_}update\text{\_}time_{i})$$

where $connection\text{\_}status_{i}$ indicates the connection status of device *i* with edge nodes or satellites, indicating whether the connection is stable, and $last\text{\_}update\text{\_}time_{i}$ represents the time when device *i* last uploaded a model update, reflecting the device’s online time and connection stability.

$\pi_{\theta }$ is specifically represented as follows:

(20) $$\pi_{\theta }(S_{i})=\arg\max_{A_{i}}Q(S_{i},A_{i})$$

where $\pi_{\theta }(S_{i})$ represents the probability or deterministic choice of policy $\pi_{\theta }$ selecting action $A_{i}$ in state $S_{i}$, $S_{i}$ represents the state information of the device, $A_{i}$ represents the device selection action, and $Q(S_{i},A_{i})$ is the Q-value function, representing the expected return after selecting action $A_{i}$ in state $S_{i}$.

The task loss function $L_{{\mathrm{task}}_{i}}(\theta )$ represents the performance of policy $\pi_{\theta }$ on task $T_{i}$, defined as follows:

(21) $$L_{{\mathrm{task}}_{i}}(\theta )=E_{S_{i}\sim S}\left[R(S_{i},A_{i})\right]$$

where E represents expectation, indicating the average return of device selection actions under the given policy $\pi_{\theta }$, $S_{i}$ represents the state (device category, bandwidth, computational capability, etc.) in task $T_{i}$, $A_{i}$ represents the action (device selection) in task $T_{i}$, and $R(S_{i},A_{i})$ is the reward function for task $T_{i}$ (return of device selection).

The goal of MAML is to minimize the loss across all tasks by updating $\theta_{\mathrm{meta}}$. The meta-loss function is defined as follows:

(22) $$L_{\mathrm{meta}}(\theta_{\mathrm{meta}})=\frac{1}{N}\sum_{i=1}^{N}L_{{\mathrm{task}}_{i}}(\theta_{\mathrm{meta}})$$

where $L_{{\mathrm{task}}_{i}}(\theta_{\mathrm{meta}})$ is the loss for task $T_{i}$, with device selection performed using the reinforcement learning policy $\pi_{\theta }$, and $\theta_{\mathrm{meta}}$ is the initial policy parameter optimized by meta-learning.

For each task $T_{i}$, the device selection strategy parameters are updated through gradient descent:

(23) $$\theta_{{\mathrm{task}}_{i}}=\theta_{\mathrm{meta}}-\alpha \nabla_{\theta }L_{{\mathrm{task}}_{i}}(\theta )$$

where α is the step size (learning rate).

The meta-parameter $\theta_{\mathrm{meta}}$ is updated as follows:

(24) $$\theta_{\mathrm{meta}}=\theta_{\mathrm{meta}}-\beta \nabla_{\theta_{\mathrm{meta}}}L_{\mathrm{meta}}(\theta_{\mathrm{meta}})$$

where β is the meta-learning learning rate, adjusting the influence of task loss on the meta-learning process.

Based on the optimized meta-parameter $\theta_{\mathrm{meta}}$, the device selection policy $\pi_{\theta_{\mathrm{meta}}}$ is updated as follows:

(25) $$\pi_{\theta_{\mathrm{meta}}}(S_{i})=\arg\max_{A_{i}}Q_{\theta_{\mathrm{meta}}}(S_{i},A_{i})$$

where $Q_{\theta_{\mathrm{meta}}}(S_{i},A_{i})$ is the Q-value function, representing the expected return after taking action $A_{i}$ in state $S_{i}$.

In new device selection tasks, the device selection policy $\theta_{\mathrm{meta}}$ is directly updated using the optimized policy parameters $\pi_{\theta_{\mathrm{meta}}}$, rapidly adjusting the policy through a small number of gradient updates to optimize device selection.

### 3.5. Privacy-Preserving Federated Learning

In the privacy-preserving federated learning process for space–air–ground integrated networks, terminal devices (such as drones, user equipment, etc.) conduct training using local data and send the resulting model parameters to edge nodes for local aggregation. Edge nodes then upload these locally aggregated results to the central server for global model aggregation.

#### 3.5.1. Local Training at Terminal Nodes

Due to the data distribution heterogeneity among drones, ground user terminals, and other devices, where each device *i*’s dataset ${𝒟}_{i}$ may have different statistical properties (such as mean, variance, etc.), data preprocessing is necessary at the local level to ensure data consistency during the training process and to reduce the impact of data heterogeneity on training results.

In space–air–ground integrated networks, sensor data collected by drones, in particular, may be subject to noise interference. Therefore, data denoising and cleaning are required. The denoising and cleaning process is represented as follows:

(26) $$x_{i,\mathrm{cleaned}}=f_{\mathrm{clean}}(x_{i})$$

where $x_{i}$ represents the original dataset on device *i*, and $f_{\mathrm{clean}}(\cdot )$ is the data cleaning function that removes noise and outliers.

Since input data $x_{i}$ from different devices may have different scales and ranges, standardization or normalization is needed to ensure numerical stability during the training process. The standardization of data $x_{i}$ is represented as follows:

(27) $$x_{i}^{\mathrm{norm}}=\frac{x_{i}-\mu_{i}}{\sigma_{i}}$$

where $\mu_{i}$ is the mean of data $x_{i}$ on device *i*, and $sigma_{i}$ is the standard deviation of data $x_{i}$ on device *i*.

The normalization of data $x_{i}$ is represented as follows:

(28) $$x_{i}^{\mathrm{scaled}}=\frac{x_{i}-x_{\min}}{x_{\max}-x_{\min}}$$

where $x_{\min}$ and $x_{\max}$ are the minimum and maximum values of data $x_{i}$ on device *i*, respectively.

In the local training phase, each device trains the model using its local dataset ${𝒟}_{i}$ with the goal of minimizing the local loss function. Assuming that device *i* uses model $f_{\theta_{i}}(\cdot )$, the objective is to update the model parameters $\theta_{i}$ by optimizing the local loss function.

Suppose the local dataset for device *i* is ${𝒟}_{i}$, the loss function $L(\theta_{i},{𝒟}_{i})$ can be expressed as

(29) $$L(\theta_{i},{𝒟}_{i})=\frac{1}{n_{i}}\sum_{(x_{j},y_{j})\in {𝒟}_{i}}L(f_{\theta_{i}}(x_{j}),y_{j})$$

where L(⋅) is the loss function on device *i*, $n_{i}$ is the number of data points on device *i*, and $\theta_{i}$ is the parameter of the model on device *i*.

Device *i* updates the model parameters $\theta_{i}$ based on the local dataset using gradient descent as follows:

(30) $$\theta_{i}^{\left(t+1\right)}=\theta_{i}^{\left(t\right)}-\eta \nabla_{\theta_{i}}L(\theta_{i}^{\left(t\right)},{𝒟}_{i})$$

where η is the learning rate used to control the step size of parameter updates. $\nabla_{\theta_{i}}L(\theta_{i}^{\left(t\right)},{𝒟}_{i})$ represents the gradient of the loss function *L*() with respect to the model parameters $\theta_{i}$, indicating the direction of steepest descent in the parameter space.

To overcome data heterogeneity and device heterogeneity, each device’s model can transfer knowledge from other devices through transfer learning. During the local training process, device *i* can dynamically select appropriate source tasks for transfer learning based on data and model similarities between devices. The mathematical model for transfer learning can be represented as

(31) $$\theta_{i}^{\mathrm{adapted}}=\theta_{s}-\eta_{\mathrm{meta}}\nabla_{\theta_{i}}L(\theta_{i},{𝒟}_{i})$$

where $\theta_{s}$ represents the model parameters transferred from other devices (source devices), and $\eta_{\mathrm{meta}}$ is the learning rate for transfer learning. Through transfer learning, device *i* can accelerate the training process with limited data by transferring knowledge (i.e., transferred model parameters) from source devices.

To protect the privacy of local data on terminal devices, differential privacy is a commonly used privacy protection method. Differential privacy ensures that individual training samples do not significantly influence the final model results by adding noise, thereby ensuring the privacy of individual data is not leaked. In privacy-preserving federated learning for space–air–ground integrated networks, each device adds differential privacy noise when uploading model parameters to prevent leakage of sensitive local data.

When device *i* uploads its local model parameters $\theta_{i}$, differential privacy noise is added to the model updates. The differential privacy noise $N_{i}$ comes from the Laplace mechanism or Gaussian mechanism, ensuring that the final uploaded model parameters do not contain information that can be directly associated with individual training data.

Assuming that device *i* has updated model parameters $\theta_{i}$ in the local training process, to ensure differential privacy, the device adds noise $N_{i}$ before uploading the local model parameters to the edge server as follows:

(32) $${\hat{\theta }}_{i}=\theta_{i}+N_{i}$$

where ${\hat{\theta }}_{i}$ is the model update with differential privacy noise, and $N_{i}$ is the noise from the Laplace distribution or Gaussian distribution, with intensity controlled by the privacy budget $\epsilon_{i}$. The $N_{i}$ based on the Laplace distribution is represented as follows:

(33) $$N_{i}\sim L(0,\frac{\Delta f_{i}}{\epsilon_{i}})$$

where $\Delta f_{i}$ is the sensitivity of the model update in device *i*’s local training process, representing the minimum response to changes in local data points. $\epsilon_{i}$ is the privacy budget of device *i*, controlling the strength of privacy protection.

One advantage of Laplace noise is that it provides strict ϵ-differential privacy, suitable for scenarios with extremely high privacy protection requirements. Compared to Gaussian noise, Laplace noise has higher computational efficiency, making it suitable for terminal devices with limited computational capabilities. One disadvantage of Laplace noise is its relatively large amplitude, which may significantly impact model performance, especially in high-dimensional data. In highly dynamic networks, larger noise may lead to slower model convergence. Unlike Laplace noise, Gaussian noise has a smaller amplitude, less impact on model performance, and is suitable for high-dimensional data and complex models. In highly dynamic networks, smaller noise helps the model converge quickly. Its weakness is that it provides weaker privacy guarantees and has a relatively higher computational complexity, which may not be friendly to terminal devices with limited computational capabilities. In this paper, when adding differential privacy noise to terminal device model updates, either Laplace noise or Gaussian noise is chosen depending on the specific application scenario to better highlight the advantages of both noise addition methods.

In space–air–ground integrated networks, device *i* may have different computational capabilities and bandwidth limitations, so the privacy budget $\epsilon_{i}$ needs to be dynamically allocated according to the characteristics of the device. For example, devices with stronger computational capabilities can be allocated a larger privacy budget, thereby reducing the impact of noise on model accuracy.

The privacy budget $\epsilon_{i}$ controls the intensity of differential privacy noise, directly affecting the privacy protection effect of the model parameters uploaded by the device and the accuracy of model training. A larger privacy budget $\epsilon_{i}$ will reduce noise, thereby improving model accuracy, but may lead to a higher risk of privacy leakage; a smaller privacy budget enhances privacy protection but may affect model accuracy. Therefore, how to dynamically allocate privacy budgets based on the device’s computational resources (such as CPU/GPU performance, memory, etc.), bandwidth, and network latency to balance privacy protection and training effectiveness is a challenge in federated learning.

The goal of this paper is to dynamically adjust the privacy budget $\epsilon_{i}$ according to device characteristics, finding a balance between privacy protection effect and training effect through optimization strategies. Based on this goal, this paper proposes a dynamic privacy budget allocation strategy based on reinforcement learning (RL) and optimization theory, aiming to optimize the privacy budget of devices through intelligent scheduling to enhance the overall efficiency of federated learning.

We view the allocation of privacy budget $\epsilon_{i}$ as a multi-objective optimization problem, considering both privacy protection and training effect. First, the privacy budget $\epsilon_{i}$ for device *i* can be represented as the following function:

(34) $$\epsilon_{i}=f(C_{i},B_{i},n_{i},\Delta f_{i})$$

where $C_{i}$ represents the computational capability of device *i* (such as CPU/GPU performance), $B_{i}$ represents the bandwidth of device *i*, $n_{i}$ represents the amount of training data on device *i*, and $\Delta f_{i}$ represents the sensitivity of the local training process on device *i*, reflecting the sensitivity of local model updates to data changes.

The objective function consists of two main aspects:

Privacy protection objective: Improve privacy protection effect, reduce the risk of privacy leakage, controlled by the standard deviation of differential privacy noise. The privacy protection objective is inversely proportional to the privacy budget $\epsilon_{i}$:

(35) $$Privacy\text{\_}Loss_{i}=\frac{1}{\epsilon_{i}}$$

Training effect objective: Improve the training accuracy of the device model. The training effect objective is related to the size of the noise; the smaller the noise, the better the training effect, usually proportional to $\epsilon_{i}$:

(36) $$Training\text{\_}Effect_{i}=\mathrm{Accuracy}(\theta_{i})\propto \epsilon_{i}$$

Combining these two objectives, the optimization objective for the privacy budget is balanced through a weighted sum approach:

(37) $${𝒪}_{i}(\epsilon_{i})=\rho_{1}\cdot Privacy\text{\_}Loss_{i}+\rho_{2}\cdot Training\text{\_}Effect_{i}$$

where $\rho_{1}$ and $\rho_{2}$ are weight coefficients used to adjust the relative importance of privacy protection and training effect.

We adopt a reinforcement learning method to dynamically adjust the privacy budget $\epsilon_{i}$ for each device, using the Q-learning algorithm to optimize the allocation of privacy budgets. The reinforcement learning strategy is defined as follows:

State space ${S_{i}}^{\prime }$: The state of device *i* consists of the device’s computational resources $C_{i}$, bandwidth $B_{i}$, training data amount $n_{i}$, and model sensitivity $\Delta f_{i}$:

(38) $${S_{i}}^{\prime }=(C_{i},B_{i},n_{i},\Delta f_{i})$$

Action space ${A_{i}}^{\prime }$: The action of device *i* is to adjust the privacy budget $\epsilon_{i}$, i.e., selecting a reasonable privacy budget value. We assume that the privacy budget can be chosen from a discrete set:

(39) $${A_{i}}^{\prime }=\{\epsilon_{i1},\epsilon_{i2},\ldots ,\epsilon_{iM}\}$$

where *M* is the number of selectable privacy budgets.

Reward function ${R_{i}}^{\prime }(\epsilon_{i})$: After selecting the privacy budget $\epsilon_{i}$, device *i* receives a reward composed of privacy protection and training effect components:

(40) $${R_{i}}^{\prime }(\epsilon_{i})=-{\lambda_{1}}^{\prime }\cdot \frac{1}{\epsilon_{i}}+{\lambda_{2}}^{\prime }\cdot \mathrm{Accuracy}(\theta_{i})$$

Optimization process: Device *i* learns the optimal allocation strategy for the privacy budget through the reinforcement learning algorithm Q-learning. The device selects the privacy budget based on its current state and updates the strategy according to the reward function ${R_{i}}^{\prime }(\epsilon_{i})$ to achieve the optimal balance between privacy protection and training accuracy.

The update formula for Q-learning to update the privacy budget allocation strategy is as follows:

(41) $$Q({S_{i}}^{\prime },\epsilon_{i})\leftarrow Q({S_{i}}^{\prime },\epsilon_{i})+\alpha \left[{R_{i}}^{\prime }(\epsilon_{i})+\gamma \max_{\epsilon_{i^{\prime }}}Q({S_{i^{\prime }}}^{\prime },\epsilon_{i^{\prime }})-Q({S_{i}}^{\prime },\epsilon_{i})\right]$$

where α is the learning rate, controlling the adaptation speed of the model to new experiences. *γ* is the discount factor, controlling the influence of future rewards.

#### 3.5.2. Local Aggregation at Edge Nodes

In space–air–ground integrated networks, terminal devices (such as UAVs, ground user terminals, etc.) train models based on local data, while edge nodes are responsible for aggregating these local models from terminal devices to form a more accurate global model. Due to the challenges faced by space–air–ground integrated networks, such as high latency, bandwidth limitations, dynamic network topology, and device heterogeneity, the role of edge nodes is crucial. Edge nodes not only need to select devices to participate in aggregation but also need to optimize the aggregation process according to device characteristics such as computational capability, bandwidth, and availability.

In privacy-preserving federated learning for space–air–ground integrated networks, edge nodes need to collect status information from terminal devices to determine which devices are suitable for participating in the current round of model aggregation. Device status information includes computational capability, bandwidth, data volume, connection status (whether stable), etc., which helps edge nodes assess device participation. The status information of device *i*, $S_{i}$, as shown in Equation (19), includes the computational capability $C_{i}$, bandwidth $B_{i}$, connection status $connection\text{\_}status_{i}$, time of last model update upload $last\text{\_}update\text{\_}time_{i}$, and other status information of device *i*. When edge nodes perform local model aggregation, they select suitable devices to participate in local model aggregation.

In privacy-preserving federated learning for space–air–ground integrated networks, due to factors such as bandwidth limitations, unstable device connections, and high communication latency, communication between devices may be interrupted, which can lead to some devices being unable to upload their locally trained model parameters to edge nodes in a timely manner. Therefore, designing an effective caching mechanism is one of the key strategies to address these issues. The caching mechanism ensures the continuity of the model and the integrity of device updates, and can cope with the instability of device connections.

After device *i* completes local training, it uploads its trained model parameters $\theta_{i}$ to the edge node. The upload depends on the device’s network connection status. If the device connection is stable, the model parameters are directly uploaded to the edge node for aggregation. When communication between the device and the edge node is interrupted (for example, periodic interruption of the connection between the satellite and the edge node), the device cannot immediately upload the model parameters. At this time, the device caches the locally trained model parameters $\theta_{i}$ until the network recovers.

The cache queue $Q_{\mathrm{cache},i}$ is used to store model parameters that have not been successfully uploaded, specifically represented as:

(42) $$Q_{\mathrm{cache},i}=\{\theta_{i,t_{1}},\theta_{i,t_{2}},\ldots ,\theta_{i,t_{k}}\}$$

where $\theta_{i,t_{k}}$ represents the cached model parameters of device *i* at time $t_{k}$. The cache queue $Q_{\mathrm{cache},i}$ stores device model updates in timestamp order.

The cache time $cache\text{\_}time_{i}$ represents the caching time of device *i* since the last upload failure, i.e., the waiting time of model parameters since the upload failure, specifically represented as

(43) $$cache\text{\_}time_{i}=t_{\mathrm{current}}-\;t_{{\mathrm{upload}}_{i}}$$

where $t_{\mathrm{current}}$ is the current time, and $t_{{\mathrm{upload}}_{i}}$ is the time when device *i* last successfully uploaded the model. The cache time directly affects device selection and model update priority. The longer the device’s cache time, the higher its upload priority.

The device’s upload priority $P_{\mathrm{priority},i}$ is dynamically scheduled based on factors such as cache time and network status, prioritizing devices with longer cache times or more stable network connections for upload, specifically represented as

(44) $$P_{\mathrm{priority},i}=\frac{\zeta_{1}\cdot cache\text{\_}time_{i}+\zeta_{2}\cdot status_{i}}{\sum_{i=1}^{N}\left(\zeta_{1}\cdot cache\text{\_}time_{i}+\zeta_{2}\cdot status_{i}\right)+\epsilon }$$

where $cache\text{\_}time_{i}$ is the cache time of device *i*, reflecting the timeliness of model upload, ${\mathrm{status}}_{i}$ represents the connection status of device *i*, giving higher priority if the device connection is stable, $\zeta_{1}$ and $\zeta_{2}$ are weight coefficients used to adjust the influence of cache time and connection status, and ϵ is a small constant used to avoid division by zero errors. The purpose of priority scheduling is to ensure that devices upload models in a reasonable order according to timeliness, avoiding delays in important model updates.

Considering that the periodic connection time interval between satellites and edge nodes is too long, which affects the accuracy of local and global models, this paper introduces a spatio-temporal redundancy mechanism to ensure that data are not lost during satellite communication interruptions by transmitting model data through other devices.

The Spatio-Temporal Redundancy Mechanism proposed in this paper refers to when communication between a specific device (such as a satellite) and an edge node is completely interrupted or experiences excessive delays, and the system activates alternative devices (such as ground base stations or neighboring devices) within proximity that have stable connections to temporarily upload cached model parameters on behalf of the disconnected device. This mechanism ensures model updates are not lost during communication interruptions by working along both spatial dimensions (utilizing reliably connected devices in geographical proximity) and temporal dimensions (preserving and delaying data transmission during communication gaps).

The spatio-temporal redundancy device set $D_{\mathrm{redundant}}$ is represented as

(45) $$D_{\mathrm{redundant}}=\{D_{j}|\mathrm{connection}(D_{j})=\mathrm{ground}\text{\_}\mathrm{and}\text{\_}\mathrm{distance}(D_{i},D_{j})\leq \delta \}$$

where δ is the maximum distance between devices, ensuring that devices have sufficient physical or communication connection capabilities for spatio-temporal redundancy.

When a device’s network connection is restored, the device uploads the cached model parameters $\theta_{i}$ according to cache time and priority scheduling. The optimization strategy for device upload is as follows:

(46) $$\theta_{i}\leftarrow \mathrm{Upload}(\theta_{\mathrm{cache},i})\;\mathrm{if}\;P_{\mathrm{priority},i}\geq \mathrm{threshold}$$

The timing of device uploads is adjusted according to the results of priority scheduling.

Edge nodes sort devices according to their priority $P_{\mathrm{priority},i}$, prioritizing devices with long cache times and stable connections for model uploads. When a device cannot connect to an edge node, a spatio-temporal redundancy device $D_{\mathrm{redundant}}$ is selected to upload the cached model. This is specifically represented as

(47) $$D_{\mathrm{selected}}=\left\{\begin{array}{ll} \arg\max_{i}P_{\mathrm{priority},i}, & status_{i}=1,\\ D_{\mathrm{redundant}}, & status_{i}=0. \end{array}\right.$$

where $D_{\mathrm{selected}}$ is the set of devices selected to participate in the upload, and the status ${\mathrm{status}}_{i}$ of a device being 1 indicates that the device has a normal connection with the edge node.

After the selected devices upload model parameters, the edge node performs local aggregation. Model aggregation is a weighted summation aggregation process based on the device’s priority and data volume. The weight $w_{i}$ of device *i* is set as a weighted combination of priority $P_{\mathrm{priority},i}$, and data volume $n_{i}$ represented as

(48) $$w_{i}=\frac{P_{\mathrm{priority},i}\cdot n_{i}}{\sum_{j\in D_{\mathrm{selected}}}P_{\mathrm{priority},j}\cdot n_{j}+\psi }$$

where $D_{\mathrm{selected}}$ is the set of devices selected to participate in the upload. $\sum_{j\in D_{\mathrm{selected}}}P_{\mathrm{priority},j}\cdot n_{j}$ is the sum of weighted priorities and data volumes of all devices participating in aggregation, which is used for normalization to ensure that the weights of all devices are between 0 and 1, avoiding excessive weight for any one device. ψ is a small constant used to avoid division by zero.

The local aggregation of model parameters uploaded by devices at the edge node is represented as

(49) $$\theta_{\mathrm{local}}=\sum_{i\in D_{\mathrm{selected}}}w_{i}\cdot \theta_{i}$$

where $w_{i}$ is the weight of device *i*, and $\theta_{i}$ is the local model parameters uploaded by device *i*.

#### 3.5.3. Global Model Aggregation at the Central Server

In privacy-preserving federated learning for integrated space–air–ground integrated networks, global aggregation at the central server is a critical step used to integrate local model updates from various edge nodes, resulting in a new global model. Before performing global aggregation, the central server receives local model updates from multiple edge nodes. These local updates are obtained after edge nodes aggregate the local model parameters uploaded from connected terminal devices (such as satellites, unmanned aerial vehicles, ground equipment, etc.).

Prior to aggregation, the central server first performs anomaly detection on the received local models to ensure that anomalous updates do not excessively influence the global model. Anomalous updates typically originate from device failures, communication issues, or malicious behavior. The server determines whether an update is anomalous by detecting differences between the model update uploaded by each edge node and those from other nodes. The anomaly detection method $A(\theta_{\mathrm{local},k})$ is expressed as follows:

(50) $$A(\theta_{\mathrm{local},k})=\left\{\begin{array}{ll} 1, & \mathrm{if}\;\|\theta_{\mathrm{local},k}-\theta_{\mathrm{avg}}{\|}_{2}>\epsilon_{\mathrm{threshold}}\\ 0, & \mathrm{otherwise} \end{array}\right.$$

where $A(\theta_{\mathrm{local},k})$ indicates whether the model update from device *k* is anomalous. This value is binary, with 1 indicating that the device’s model update is considered anomalous, and 0 indicating that the device’s model update is normal. $\theta_{\mathrm{local},k}$ represents the local model update uploaded by device *k*. $\|\cdot {\|}_{2}$ represents the Euclidean distance, measuring the degree of difference between the local model update $\theta_{\mathrm{local},k}$ from device *k* and the global average model update $\theta_{\mathrm{avg}}$. This distance measures the degree to which the local update deviates from the global model; the larger it is, the more likely the model update uploaded by the device is anomalous. $\epsilon_{\mathrm{threshold}}$ is the anomaly detection threshold, used to determine whether a model update deviates beyond the acceptable range from the global model. $\theta_{\mathrm{avg}}$ is the weighted average of all model updates uploaded by devices participating in federated learning. This value serves as a reference benchmark for the global model and as a basis for determining whether a device’s model update is anomalous. The calculation formula for $\theta_{\mathrm{avg}}$ is as follows:

(51) $$\theta_{\mathrm{avg}}=\frac{1}{K}\sum_{k=1}^{K}\theta_{\mathrm{local},k}$$

where *K* is the number of devices participating in the aggregation.

Once anomalies are detected, the central server employs robust aggregation techniques to handle anomalous updates. Compared to the weighted average method, the clipping method can effectively prevent anomalous updates from affecting the global model, especially when there are significant differences in model updates, thereby ensuring the robustness of the global model. The model anomaly update processing based on the clipping method is expressed as follows:

(52) $$\theta_{\mathrm{local},k}^{\mathrm{trimmed}}=\mathrm{clip}(\theta_{\mathrm{local},k},\theta_{\min},\theta_{\max})$$

where $\theta_{\mathrm{local},k}^{\mathrm{trimmed}}$ represents the corrected local model update, aimed at rectifying anomalous updates to bring them within a reasonable range. $\theta_{\mathrm{local},k}$ represents the original local model update uploaded by device *k*. $\mathrm{clip}(\cdot ,\theta_{\min},\theta_{\max})$ is the clipping operation, which restricts the model update values within a specified range. Specifically, if the value of $\theta_{\mathrm{local},k}$ exceeds the upper and lower limits $\theta_{\max}$ and $\theta_{\min}$, it will be adjusted to these limit values. $\theta_{\max}$ and $\theta_{\min}$ are the set maximum and minimum values allowed for model updates, which are determined by calculating the standard deviation of all device-uploaded model updates.

Assuming that the model $\theta_{\mathrm{local},k}$ uploaded by each device has a certain distribution range, a reasonable clipping range can be defined by calculating the standard deviation of the model parameters, specifically expressed as follows:

(53) $$\sigma =\sqrt{\frac{1}{K}\sum_{k=1}^{K}\|\theta_{\mathrm{local},k}-\theta_{\mathrm{avg}}{\|}_{2}^{2}}$$

where *K* is the number of edge devices participating in aggregation, $\theta_{\mathrm{local},k}$ represents the local model update uploaded by device *k*, $\theta_{\mathrm{avg}}$ is the weighted average of all model updates uploaded by devices participating in federated learning, and $\|\cdot {\|}_{2}$ represents the Euclidean distance.

The minimum value allowed for model updates $\theta_{\min}$ is set as follows:

(54) $$\theta_{\min}=\theta_{\mathrm{avg}}-3\sigma$$

The maximum value allowed for model updates $\theta_{\max}$ is set as follows:

(55) $$\theta_{\max}=\theta_{\mathrm{avg}}+3\sigma$$

Here, three times the standard deviation is used as the upper and lower limits. Based on statistical principles, assuming that model updates follow a normal distribution, three times the standard deviation covers most reasonable model updates.

Considering that in typical situations, some edge nodes may be anomalous while others are not, to facilitate global model aggregation, ${\theta_{\mathrm{local},k}}^{\prime }$ is used to represent the unified local model update of edge nodes, specifically expressed as follows:

(56) $${\theta_{\mathrm{local},k}}^{\prime }=\left\{\begin{array}{ll} \theta_{\mathrm{local},k}^{\mathrm{trimmed}}, & \mathrm{if}\;A(\theta_{\mathrm{local},k})=1\\ \theta_{\mathrm{local},k}, & \mathrm{otherwise} \end{array}\right.$$

In privacy-preserving federated learning for integrated space–air–ground integrated networks, the weight $w_{k}$ of edge node *k* is used to measure the importance or contribution of each edge node in global model aggregation. Weight calculation primarily depends on the device’s data volume and priority, ensuring that devices with better network quality and larger data volumes can make greater contributions to the global model update during model aggregation. The calculation formula for $w_{k}$ is as follows:

(57) $$w_{k}=\frac{P_{\mathrm{priority},k}\cdot n_{k}}{\sum_{i=1}^{K}P_{\mathrm{priority},i}\cdot n_{i}+\epsilon_{w}}$$

where *K* is the number of edge devices participating in aggregation, $n_{k}$ represents the data volume uploaded by edge node *k*, $\epsilon_{w}$ is a small constant used to prevent division-by-zero errors, and $P_{\mathrm{priority},k}$ represents the upload priority of edge node *k*.

The calculation of priority $P_{\mathrm{priority},k}$ depends more on the device’s network stability, latency, and data volume, with the calculation formula as follows:

(58) $$P_{\mathrm{priority},k}=\frac{n_{k}\cdot (1-p_{k}^{\mathrm{loss}})}{d_{k}+\epsilon_{P}}$$

where $n_{k}$ represents the data volume uploaded by device *k*, which is the size of the model update uploaded by that device. $p_{k}^{\mathrm{loss}}$ is the packet loss rate of device *k*, indicating the quality of the network connection between the device and the edge node. $d_{k}$ represents the communication delay between device *k* and the central server, and $\epsilon_{P}$ is a constant to prevent division by zero.

In privacy-preserving federated learning for integrated space–air–ground integrated networks, global model aggregation is the core step of the entire federated learning process. The purpose of global aggregation is to aggregate local model updates uploaded from various devices into a global model for use in the next round of training. Global model aggregation weights the local updates of each device according to its contribution. The global model aggregation formula is as follows:

(59) $$\theta_{\mathrm{global}}^{t+1}=\sum_{k=1}^{K}w_{k}\cdot {\theta_{\mathrm{local},k}}^{\prime }+\lambda_{\theta }\cdot \Delta_{\mathrm{domain}}$$

where $w_{k}$ represents the weight of edge node *k*, ${\theta_{\mathrm{local},k}}^{\prime }$ represents the local model update of edge node *k*, $\lambda_{\theta }$ is the cross-domain transfer correction coefficient used to adjust the influence of cross-device differences on the global model, and $\Delta_{\mathrm{domain}}$ is the cross-domain transfer correction term used to mitigate data distribution differences between devices. $\Delta_{\mathrm{domain}}$ is defined as follows:

(60) $$\Delta_{\mathrm{domain}}=\frac{1}{K-1}\sum_{j\neq k}\|\theta_{\mathrm{local},k}-\theta_{\mathrm{local},j}{\|}_{2}^{2}$$

where $\theta_{\mathrm{local},j}$ and $\theta_{\mathrm{local},k}$ are the local models of different edge devices *j* and *k*.

$\Delta_{\mathrm{domain}}$ measures the model update differences between devices, reducing problems caused by data distribution differences between devices, making the global model aggregation more stable and consistent.

## 4. Experimental Evaluation

To verify the effectiveness of the proposed Privacy-Preserving Federated Learning method for Space–Air–Ground Integrated Networks (PPFL-SAGINs), we compared it with existing methods and analyzed the experimental results. First, we introduce the experimental setup and datasets, followed by experimental verification of the proposed method across different datasets and dimensions.

### 4.1. Experimental Setup

#### 4.1.1. Experimental Environment

The simulation experiments were developed in PyCharm 17.0.10, with Python 3.9 as the programming language and PyTorch 2.3 as the deep learning framework. The hardware configuration was as follows: Intel(R) Core(TM) i7-7820HQ CPU @ 2.90 GHz, 32 GB RAM, NVIDIA Quadro P5000 GPU with 16 GB video memory, and 2 TB HDD.

The simulation of the Space–Air–Ground Integrated Network system included 10 terminal nodes, 3 edge nodes, and 1 central server. Among these, 4 terminal nodes were located in the ground layer, directly connected to edge devices; 3 terminal nodes were in the aerial layer; and 3 terminal nodes communicated with edge nodes via satellite. Appendix A shows the code structure of the simulation experiment.

Table 1 shows the dynamic topology parameters. The simulation dynamically adjusts these parameters throughout the training process to evaluate the robustness of our proposed framework under various network conditions. Particularly, we programmed specific topology change events, such as periodic LEO satellite connectivity interruptions, UAV flight path variations, and ground device mobility, to test the framework’s resilience to SAGINs’ dynamic nature.

#### 4.1.2. Datasets

The experiments were conducted using the MNIST [36] dataset, the EuroSAT dataset [33], UC Merced Land Use Dataset [37], and RSI-CB dataset [38].

The MNIST dataset contains handwritten digit images from 0 to 9. It is a standard benchmark dataset widely used to evaluate the performance of various algorithms. It comprises 10 categories, with each image being 28 × 28 pixels.

The EuroSAT dataset is a remote sensing dataset based on satellite imagery, containing 10 different land use types (such as agriculture, forest, water bodies, etc.). Each image is 64 × 64 pixels.

The UC Merced Land Use dataset contains high-resolution remote sensing images commonly used for land use classification and remote sensing image analysis. It includes 21 categories, such as agricultural land, urban areas, forests, lakes, roads, etc. The dataset contains 2100 images, with 100 images per category. Each image is 256 × 256 pixels.

The RSI-CB (Remote Sensing Image Classification Benchmark) dataset is a large-scale remote sensing image classification benchmark dataset constructed based on crowdsourced data. This dataset aims to provide better training and testing resources for deep learning in the field of remote sensing image classification. The RSI-CB dataset contains two subsets, RSI-CB256 and RSI-CB128. RSI-CB256 contains 35 categories with approximately 24,000 images, while RSI-CB128 contains 45 categories with 36,707 images of 128 × 128 pixels. This paper conducts validation based on RSI-CB128.

While these datasets are standard benchmarks in machine learning, they were specifically selected to simulate real-world AI applications in SAGIN environments. The MNIST dataset, though a general image classification benchmark, represents the type of lightweight classification tasks that might be performed by resource-constrained devices at various SAGIN layers, such as object recognition by UAVs or pattern detection on satellite imagery. More directly relevant to SAGIN applications, the EuroSAT, RSI-CB and UC Merced Land Use datasets represent actual remote sensing tasks performed by satellites and aerial platforms in real-world scenarios. These datasets simulate critical SAGIN-dependent AI applications mentioned earlier (line 131), including disaster monitoring, environmental surveillance, and precision agriculture, where satellite imagery analysis must be conducted across distributed nodes with privacy concerns. The heterogeneous nature of these datasets also allows us to evaluate our framework’s performance on tasks with varying complexity and data distributions, mirroring the diverse AI workloads in SAGIN environments where computation must be coordinated across space, air, and ground layers while preserving data privacy.

### 4.2. Experimental Results and Analysis

To better compare the effectiveness of the proposed Privacy-Preserving Federated Learning method for Space–Air–Ground Integrated Networks (PPFL-SAGINs), we compared it with existing federated learning methods including FedAvg [16], Asynchronous Federated Learning (FedAsync) [39], and Two-Tier Federated Learning (FedAsyncISL) [40]. To ensure the accuracy of the experimental results, the data presented in this paper are the average values obtained from three runs.

#### 4.2.1. Performance Metric Evaluation Based on Four Datasets

To evaluate the performance of the proposed Privacy-Preserving Federated Learning method (PPFL-SAGIN) in different scenarios, we validated it through image recognition tasks using the MNIST dataset, the EuroSAT dataset, and the UC Merced Land Use dataset.

**Figure 4:**
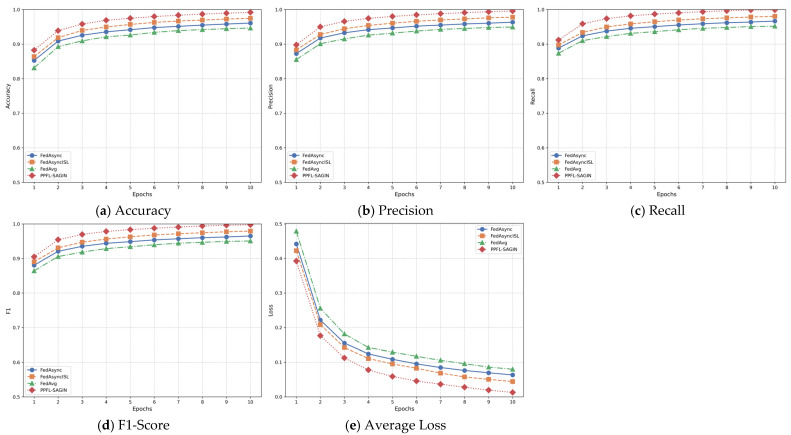
Performance of PPFL-SAGIN, FedAvg, FedAsync, and FedAsyncISL based on the MNIST dataset.

Figure 4 shows the performance of the PPFL-SAGIN, FedAvg, FedAsync, and FedAsyncISL based on the MNIST dataset.

**Figure 5:**
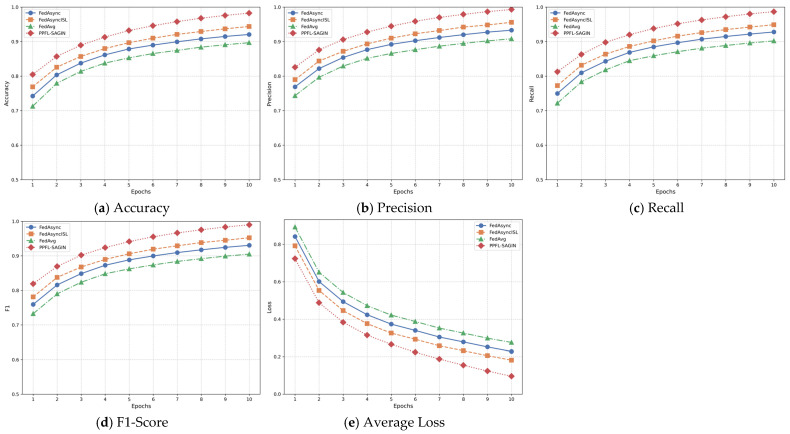
Performance of PPFL-SAGIN, FedAvg, FedAsync, and FedAsyncISL based on the EuroSAT dataset.

Figure 5 shows the performance of PPFL-SAGIN, FedAvg, FedAsync, and FedAsyncISL based on the EuroSAT dataset.

**Figure 6:**
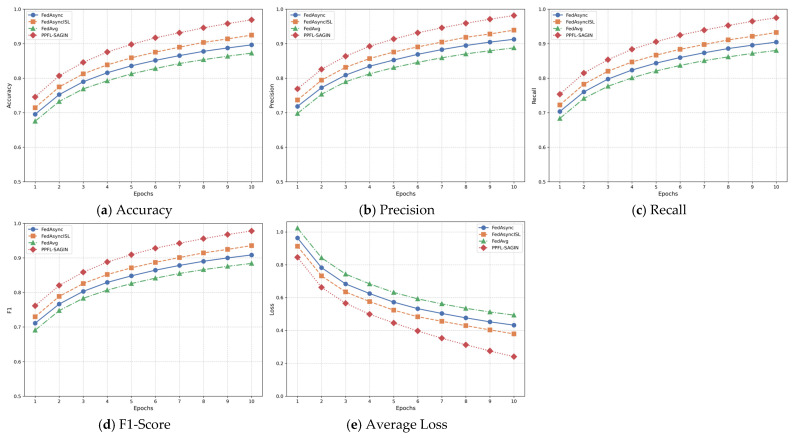
Performance of PPFL-SAGIN, FedAvg, FedAsync, and FedAsyncISL based on the UC Merced Land Use dataset.

Figure 6 shows the performance of PPFL-SAGIN, FedAvg, FedAsync, and FedAsyncISL based on the UC Merced Land Use dataset.

**Figure 7:**
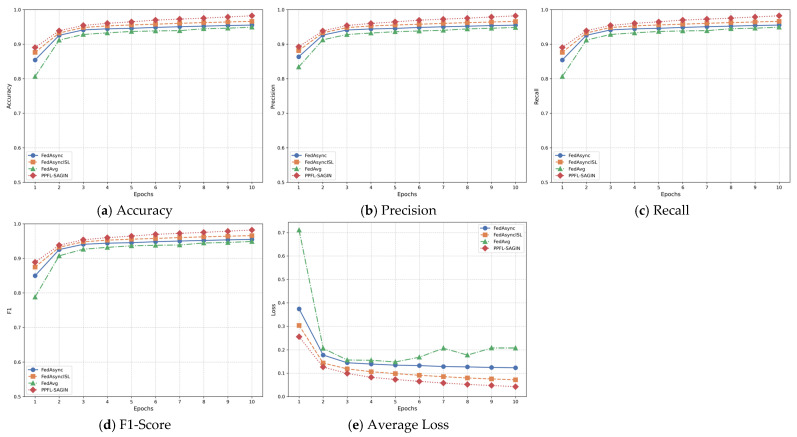
Performance of PPFL-SAGIN, FedAvg, FedAsync, and FedAsyncISL based on the RSI-CB dataset.

Figure 7 shows the performance of PPFL-SAGIN, FedAvg, FedAsync, and FedAsyncISL based on the RSI-CB dataset.

Figure 4a, Figure 5a, Figure 6a and Figure 7a illustrate the accuracy performance of the PPFL-SAGIN, FedAvg, FedAsync, and FedAsyncISL on the MNIST, EuroSAT, UC Merced Land Use, and RSI-CB datasets. It is evident that the PPFL-SAGIN achieved the highest accuracy across all rounds and datasets, with FedAsyncISL consistently ranking second, its accuracy values lying between those of the PPFL-SAGIN and the standard FedAsync method. FedAsync outperformed FedAvg but could not reach the level of the PPFL-SAGIN or FedAsyncISL. In terms of convergence, the PPFL-SAGIN not only achieved higher accuracy but also converged faster, especially in the early rounds, where its learning curve was steeper. All methods exhibited diminishing marginal returns after approximately 6–7 rounds, with the accuracy curves flattening, though the PPFL-SAGIN maintained its advantage. The consistent superiority of the PPFL-SAGIN across multiple datasets indicates that its approach to handling asynchronous updates and client selection provides significant advantages in federated learning scenarios.

Figure 4b, Figure 5b, Figure 6b and Figure 7b illustrate the precision performance of the PPFL-SAGIN, FedAvg, FedAsync, and FedAsyncISL on the MNIST, EuroSAT, UC Merced Land Use and RSI-CB datasets. The PPFL-SAGIN achieved the highest precision across all rounds and datasets, followed by FedAsyncISL, which significantly outperformed the basic asynchronous method. FedAsync performed better than the traditional federated averaging method, while FedAvg exhibited the lowest performance in all scenarios. In the early training stages (rounds 1–3), the PPFL-SAGIN demonstrated significantly higher precision compared to other methods, indicating that its adaptive knowledge sharing mechanism can quickly adapt to data distribution differences among different devices, accelerating model convergence. Maintaining a stable lead across four datasets of varying complexity demonstrates that its dynamic weighting factors effectively balance device heterogeneity and data distribution differences, enabling the model to perform excellently in different application scenarios. In later rounds (6–10), the PPFL-SAGIN continued to show an upward trend while the other methods plateaued, suggesting that its knowledge sharing mechanism can continuously discover and integrate valuable features, avoiding premature convergence to local optima.

Figure 4c, Figure 5c, Figure 6c and Figure 7c illustrate the recall curves for the PPFL-SAGIN, FedAvg, FedAsync, and FedAsyncISL on the MNIST, EuroSAT, UC Merced Land Use, and RSI-CB datasets. The PPFL-SAGIN performed best across all datasets and training rounds, with FedAsyncISL ranking second but showing a significant gap compared to the PPFL-SAGIN. FedAsync outperformed the traditional FedAvg method, while FedAvg showed the weakest performance, particularly on complex datasets. Regarding adaptability to heterogeneous environments, the PPFL-SAGIN effectively handled data distribution differences among different devices through dynamic weighting factors, maintaining a high rate of recall improvement across all datasets. This advantage was particularly evident in the more complex EuroSAT and UC Merced Land Use datasets. In terms of knowledge transfer efficiency, especially in the initial rounds (1–3), the PPFL-SAGIN quickly established a high recall baseline, verifying that its knowledge sharing mechanism effectively integrates knowledge from different devices in the early stages of training, avoiding the problem of traditional methods requiring a longer time to form effective representations.

Figure 4d, Figure 5d, Figure 6d and Figure 7d illustrate the F1-score curves for the PPFL-SAGIN, FedAvg, FedAsync, and FedAsyncISL on the MNIST, EuroSAT, UC Merced Land Use, and RSI-CB datasets. The PPFL-SAGIN method performed best on all four datasets, maintaining a leading advantage from the first epoch and consistently maintaining the highest F1-score throughout the training process. The PPFL-SAGIN not only achieved the highest final performance but also converged significantly faster than other methods. This advantage was particularly pronounced in the more complex EuroSAT and UC Merced Land Use datasets. The two-tier reinforcement learning device selection strategy adopted by the PPFL-SAGIN, combined with meta-learning and hierarchical attention mechanisms, significantly improved the convergence efficiency of the model.

Figure 4e, Figure 5e, Figure 6e and Figure 7e illustrate the average loss curves for the PPFL-SAGIN, FedAvg, FedAsync, and FedAsyncISL on the MNIST, EuroSAT, UC Merced Land Use, and RSI-CB datasets. In terms of convergence performance, the SAGIN method exhibited the optimal loss convergence curve across all three datasets, with the lowest final loss value. Notably, on all four datasets, the initial loss (at the first epoch) of the PPFL-SAGIN was lower than that of the other three methods, indicating that this method can effectively utilize the adaptive knowledge sharing mechanism of transfer learning to quickly adapt to data distribution from the early stages of training. The PPFL-SAGIN also demonstrated a faster rate of loss decline, especially in the first 5 epochs of training, with a slope of decline significantly greater than other methods, reflecting the efficiency of its optimization strategy.

#### 4.2.2. Time Overhead Evaluation

In this paper, time overhead refers to the total computational time required to complete the entire federated learning process, measured in seconds. It includes the time for local model training on devices, model parameter transmission between devices and edge nodes, local aggregation at edge nodes, and global model aggregation at the central server.

**Figure 8:**
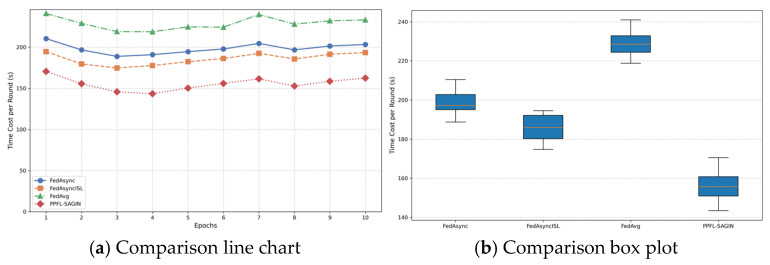
Training time overhead comparison based on the MNIST dataset.

**Figure 9:**
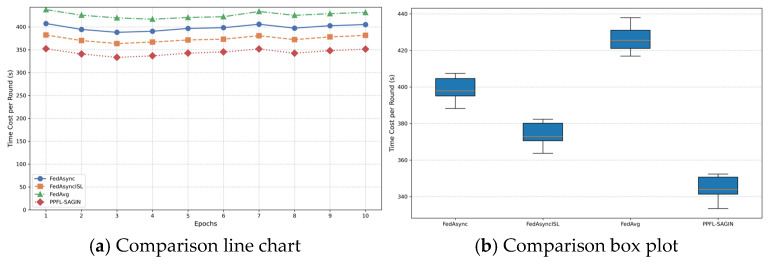
Training time overhead comparison based on the UC Merced Land Use dataset.

We compared the time overhead of the four methods based on the MNIST dataset and the UC Merced Land Use dataset, with the specific results shown in Figure 8 and Figure 9.

From Figure 8a, it is evident that FedAvg consistently maintained the highest time cost across all rounds, with FedAsync showing the second-highest time cost. FedAsyncISL performed better than FedAsync, and compared to the above three methods, the PPFL-SAGIN had the lowest time cost in all rounds. Figure 8b indicates that FedAvg had the highest median time and a wider interquartile range (IQR), suggesting greater variability. FedAsync had a median time of approximately 195–200 s with a moderate IQR. FedAsyncISL had a median time of approximately 185 s with a moderate IQR. The PPFL-SAGIN had the lowest median time with a relatively compact IQR, indicating more stable performance. The box plot confirmed that the PPFL-SAGIN was not only faster but also exhibited more stable performance compared to other algorithms.

Figure 9 compares the time overhead of the four methods over 10 rounds based on the UC Merced Land Use dataset. From Figure 9a, it is clear that FedAvg had the highest overall time overhead, slightly higher in the first round and rounds 7–10. FedAsync had the second-highest time overhead, showing a decreasing trend in the early rounds and a slight increase in the later rounds. FedAsyncISL had the third-highest time overhead, exhibiting a more obvious “U-shaped” trend. The PPFL-SAGIN had the lowest time overhead, with a trend similar to that of FedAsyncISL. As shown in Figure 9b, FedAvg had a median time overhead of approximately 425 s with a relatively small fluctuation range. FedAsync had a median time overhead of 398 s with moderate fluctuation. FedAsyncISL had a median time overhead of approximately 374 s with a relatively compact distribution. The PPFL-SAGIN had a median time overhead of approximately 344 s with the smallest fluctuation. Overall, compared to the other three methods, the PPFL-SAGIN had the lowest time overhead and good stability.

#### 4.2.3. Privacy Protection Evaluation

**Figure 10:**
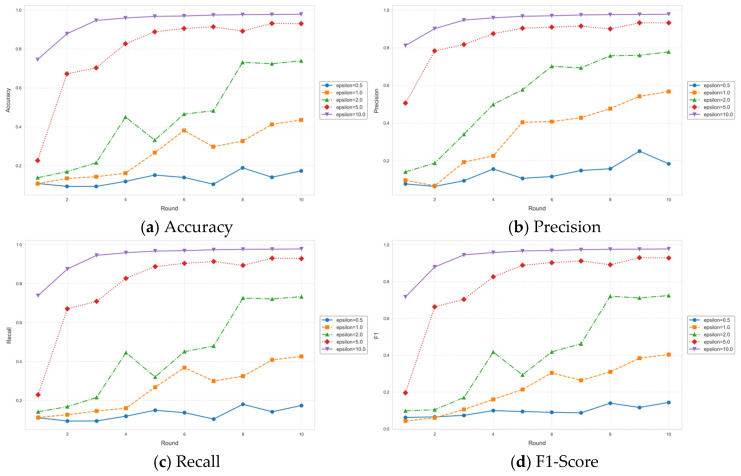
Comparison of the impact of different privacy budgets on model accuracy based on the MNIST dataset.

Figure 10 shows the comparison results of the impact of different privacy budgets on model accuracy based on the MNIST dataset.

Figure 10 illustrates the comparison of Accuracy, Precision, F1-Score, and Recall metrics for federated learning in space–air–ground integrated networks under different privacy budget values based on the MNIST dataset. The experimental results clearly demonstrate the trade-off between performance and privacy protection strength: the larger the privacy budget ε, the higher the performance metrics generally are, but the corresponding privacy protection strength decreases. In terms of convergence speed, higher privacy budgets (ε = 5.0 and ε = 10.0) exhibited significantly faster convergence rates, with rapid performance improvements in the first 3 iterations. In contrast, low privacy budget settings (ε = 0.5 and ε = 1.0) showed slow and unstable convergence processes. The ε = 10.0 configuration reached an accuracy of approximately 98% by the 10th round, while the ε = 0.5 configuration only achieved an accuracy of about 18%, indicating that the model struggles to effectively learn data distributions under extremely low privacy budgets.

As can be observed from the figure, there are noticeable fluctuations in the performance metrics at lower privacy budget values (such as ε = 0.5 and ε = 1.0). This phenomenon primarily stems from the inverse relationship between privacy budget and noise interference—smaller privacy budgets necessitate larger noise addition, resulting in more unstable gradient information during model updates. Additionally, the exploration behavior inherent in the dual-layer reinforcement learning strategy occasionally leads the system to investigate new device combinations across certain rounds, causing temporary performance variations. While these exploratory actions are essential for long-term optimization, they inevitably introduce short-term fluctuations in performance metrics.

#### 4.2.4. Ablation Study

This ablation study aims to validate the effectiveness of the dynamic weighting strategy in the PPFL-SAGIN method proposed in this paper, particularly its performance in environments with severe data heterogeneity. The experiment was conducted using the MNIST dataset, where the satellite layer primarily contains samples from classes 0–3 with proportions of 50%, 30%, 15%, and 5%, respectively; the aerial layer primarily contains samples from classes 4–6 with proportions of 45%, 35%, and 20%; and the ground layer primarily contains samples from classes 7–9 with proportions of 40%, 35%, and 25%. In terms of data volume distribution, satellite nodes have limited resources, aerial nodes have moderate resources, and ground nodes have abundant resources. Regarding device heterogeneity, the satellite layer has low computing capability with strict bandwidth limitations (1–5 Mbps), the aerial layer has moderate computing capability with moderate bandwidth limitations, and the ground layer has high computing capability with sufficient bandwidth. Table 2 shows the performance metrics of the PPFL-SAGIN (dynamic weighting) and the method based on static weighting strategy after 10 rounds of training.

As shown in Table 2, the dynamic weighting strategy achieved significant improvements across all evaluation metrics, particularly with a 55.32% reduction in average loss, indicating a substantial improvement in model fitting quality. In terms of convergence speed, the PPFL-SAGIN (dynamic weighting) achieved over 90% accuracy by the 6th round, while the static weighting strategy failed to reach 90% accuracy within 10 rounds.

By analyzing the accuracy of different methods across various categories, the following results were obtained: The static weighting strategy achieved 67–75% accuracy for satellite layer categories (0–3), 84–88% accuracy for aerial layer categories (4–6), and 92–95% accuracy for ground layer categories (7–9), showing excessive dependence on ground layer nodes with larger data volumes and poorer fitting for satellite layer categories. The PPFL-SAGIN (dynamic weighting) method achieved 91–94% accuracy for satellite layer categories (0–3), 94–96% accuracy for aerial layer categories (4–6), and 95–97% accuracy for ground layer categories (7–9), demonstrating more balanced accuracy across categories and good fitting even for satellite layer categories with fewer samples.

The experimental results indicate that the dynamic weighting factor design proposed in Equation (1) of this paper is crucial for addressing data heterogeneity issues in space–air–ground integrated networks, effectively handling complex, heterogeneous network environments while ensuring model performance.

## 5. Conclusions

The privacy-preserving federated learning optimization strategy for space–air–ground integrated networks proposed in this paper successfully addresses the challenges of efficient device selection, privacy protection, and model aggregation in complex space–air–ground integrated network environments. By combining two-tier reinforcement learning, transfer learning, hierarchical attention mechanisms, and meta-learning-based strategy optimization, this method effectively copes with dynamic topologies, latency, bandwidth limitations, and device heterogeneity in the network, enhancing the efficiency and flexibility of device selection. Additionally, the differential privacy-based privacy protection mechanism ensures that data privacy is not compromised, enhancing the security of the model. Experimental results validate the efficiency and feasibility of this method in multi-task environments.

Future work will consider further research on handling anomalous nodes in the global aggregation phase and proposing more efficient robust aggregation algorithms. Through further investigation of these issues, it is expected that more efficient, stable, and secure solutions for federated learning in space–air–ground integrated networks can be provided.

## Figures and Tables

**Table 1:** Dynamic topology parameters.

Parameter	Value/Range	Description
		LEO: Circular orbit with 90-min period
		MEO: Circular orbit with 6-h period
Node Mobility Pattern	Varied	GEO: Fixed relative to Earth
		UAV: Random waypoint model at 100–300 m
		HAPS: Fixed position at 20 km altitude
		LEO–Ground: 5–15 min
		MEO–Ground: 20–40 min
Connection Duration(min)	5—Continuous	GEO–Ground: Continuous
		UAV–Ground: 10–30 min
		HAPS–Ground: Continuous
		LEO visibility changes: Every 5–15 min
Topology Change Frequency	Varied	UAV connectivity changes: Every 10–30 min
		Ground device mobility: Every 30–60 min
		Space nodes: 10–50%
Disconnection Probability	0–50%	Air nodes: 5–30%
(per hour)		Ground nodes: 0–10%

**Table 2:** Comparison of performance metrics for two methods after 10 rounds of training.

Method	Accuracy	Precision	Recall	F1-Score	Average Loss
PPFL-SAGIN (dynamic weighting)	0.9502	0.9534	0.9502	0.9512	0.1204
Static weighting strategy	0.8512	0.8567	0.8512	0.8525	0.2695

## Data Availability

Data are contained within the article.

## Appendix A

Experimental Code Structure and Explanation.

The codebase is structured as follows:

├── main.py # Main entry point for experiments

├── config/

│ └── config.yaml # Configuration parameters

├── models/

│ ├── neural_networks.py # Neural network model definitions

│ └── federated_models.py # Federated learning model implementations

├── algorithms/

│ ├── adaptive_transfer.py # Adaptive transfer learning implementation

│ ├── hierarchical_rl.py # Hierarchical reinforcement learning

│ ├── privacy_budget.py # Privacy budget allocation

│ └── robust_aggregation.py # Robust aggregation methods

├── utils/

│ ├── data_loader.py # Dataset loading and preprocessing

│ ├── metrics.py # Evaluation metrics

│ └── visualization.py # Results visualization

└── simulation/

├── network_topology.py # SAGIN network topology simulator

├── device_manager.py # Device status and capability simulator

  └── communication.py # Communication channel simulator

Detailed Code Structure Introduction

main.py

main.py serves as the primary entry point for experiments, responsible for initializing the entire system and controlling the execution flow. This file implements the following functionalities: parsing configuration files and initializing the experimental environment, creating and configuring the space–air–ground integrated network simulation environment, initializing terminal devices, edge nodes, and the central server, and coordinating the federated learning training process, including local training, model aggregation, and evaluation.

config/

The config/folder contains experimental configuration parameters, primarily including config.yaml, which stores experiment parameters such as network topology parameters, federated learning parameters, differential privacy parameters, device parameter configurations, and dataset configurations.

models/

The models/folder contains neural network model definitions and federated learning model implementations. Specifically, neural_networks.py implements the neural network models used in experiments, while federated_models.py implements various federated learning algorithms, including FedAvg, FedAsync, FedAsyncISL, and others.

algorithms/

The algorithms/folder contains implementations of the core algorithms proposed in this paper. Among them, adaptive_transfer.py implements the adaptive transfer learning mechanism, including dynamic weighting factor calculation, task matching degree calculation, and transfer learning model updates. hierarchical_rl.py implements the device selection strategy based on hierarchical reinforcement learning, including global layer decision-making, local layer decision-making, and meta-learning-based strategy optimization. privacy_budget.py implements the privacy budget allocation strategy, including Laplace and Gaussian noise mechanisms, dynamic privacy budget allocation, and reinforcement learning-based privacy budget optimization.

utils/

The utils/folder contains utility functions, including data_loader.py which implements dataset loading and preprocessing, metrics.py which implements evaluation metric calculations such as accuracy, precision, recall, and F1-score, and visualization.py which implements result visualization.

simulation/

The simulation/folder contains the implementation of the space–air–groundintegrated network simulation, where network_topology.py serves as the network topology simulator, device_manager.py is the device status and capability simulator, and communication.py is the communication channel simulator.
